# Divergence in the metabolome between natural aging and Alzheimer’s disease

**DOI:** 10.1038/s41598-020-68739-z

**Published:** 2020-07-22

**Authors:** Holly C. Hunsberger, Bennett P. Greenwood, Vladimir Tolstikov, Niven R. Narain, Michael A. Kiebish, Christine Ann Denny

**Affiliations:** 1 Division of Systems Neuroscience, Research Foundation for Mental Hygiene, Inc. (RFMH)/New York State Psychiatric Institute (NYSPI), New York, NY USA; 2 Department of Psychiatry, Columbia University Irving Medical Center (CUIMC), NYSPI Kolb Research Annex, Room 777, 1051 Riverside Drive, Unit 87, New York, NY USA; 3 BERG, Framingham, MA USA

**Keywords:** Neuroscience, Biomarkers, Diseases

## Abstract

Alzheimer’s disease (AD) is a progressive and debilitating neurodegenerative disorder and one of the leading causes of death in the United States. Although amyloid plaques and fibrillary tangles are hallmarks of AD, research suggests that pathology associated with AD often begins 20 or more years before symptoms appear. Therefore, it is essential to identify early-stage biomarkers in those at risk for AD and age-related cognitive decline (ARCD) in order to develop preventative treatments. Here, we used an untargeted metabolomics analysis to define system-level alterations following cognitive decline in aged and APP/PS1 (AD) mice. At 6, 12, and 24 months of age, both control (Ctrl) and AD mice were tested in a 3-shock contextual fear conditioning (CFC) paradigm to assess memory decline. AD mice exhibited memory deficits across age and these memory deficits were also seen in naturally aged mice. Prefrontal cortex (PFC), hippocampus (HPC), and spleen were then collected and analyzed for metabolomic alterations. A number of significant pathways were altered between Ctrl and AD mice and naturally aged mice. By identifying systems-level alterations following ARCD and AD, these data could provide insights into disease mechanisms and advance the development of biomarker panels.

## Introduction

Alzheimer’s disease (AD), a progressive and debilitating neurodegenerative disorder, characterized by senile amyloid plaques and tau fibrillary tangles, is the leading cause of dementia^1^. Amyloid plaques are aggregates of various amyloid peptides derived from the amyloid precursor protein (APP). APP accumulates when presenilin 1 and 2 mutant genes (PS1 and PS2) enhance the γ-secretase-mediated processing of APP^2^. Therefore, we have chosen the APP/PS1 AD mouse model to best replicate the human amyloid cascade. This amyloid accumulation then promotes the spread of tau tangles, consisting of hyperphosphorylated tau protein, inevitably leading to cognitive decline^3^. However, research suggests that the pathology associated with AD often begins 20 or more years before symptoms appear^4^, which makes this a critical time window for possible preventative treatments.

Because aging is the greatest risk factor for developing AD^5^, it is also important to examine age-related brain changes and age-related cognitive decline (ARCD). ARCD is a normal process of aging where conceptual reasoning, memory, and processing speed gradually decline over time (fluid intelligence)^6–8^. However, ARCD differs from AD in that certain abilities, such as vocabulary or other skills that have accumulated throughout life, are resilient to brain aging (crystallized intelligence)^9^. It is important to understand how metabolites influence ARCD and AD, as our current aging population is expected to double in the next 40 years^10^.

One of the biggest problems facing AD research is the lack of biomarkers for diagnosis and treatment. Currently, there are only 3 FDA-approved diagnostic tests for AD, all of which are positron emission tomography (PET) neuroimaging scans for amyloid^11–13^. Although several tau PET tracers are used actively in research (i.e., F-FDDNP, C-PBB3, FTP)^14^, the FDA has not yet approved these tracers for clinical diagnosis^15^. Moreover, these scans are invasive, costly, and are not definitive. The FDA also approved use of an at-home genetic test (commonly known as *23andMe*) for the *APOE4* gene variant, which is a risk factor for AD^16^. However, this risk is inconsistent in minority populations, such as African Americans and Hispanics, and between males and females^17,18^. Interestingly, metabolic decline is one of the earliest symptoms in patients with mild cognitive impairment (MCI), defined by cognitive decline that does not result in mood changes or a change in independent living, as detected by PET^19^. MCI affects 19% of people 65 and older and depending on the sample, 46% of people with MCI will transition to dementia^20^. This suggests that metabolism could play a role early in the disease process.

Metabolomics, used to measure levels of small molecule metabolites in biological samples, represents one of the best omics platforms for the diagnosis and prognosis of sporadic AD, as the metabolome is a reflection of genetics, protein profiles, and environmental influences^21–23^. Sporadic AD is a multifaceted disease with multiple biological pathways affected^24^. For example, in MCI patients, lysine metabolism, the tricarboxylic acid cycle (TCA) cycle, lipid metabolism, and mitochondrial dysfunction are perturbed early in the disease compared to healthy individuals both in plasma and cerebral spinal fluid (CSF)^21^. Many of these results are also observed in mouse models of AD. Specifically, Gonzalez-Dominguez and colleagues analyzed tissue and serum in the APP/PS1 mice and found that at 6 months of age, metabolic changes in fatty acids and phospholipids were observed in the HPC and cortex, while inflammatory and immune pathways were heavily altered in plasma^25,26^. However, although these reports provided a comprehensive metabolomic dataset, these studies only examined metabolites at one time point.

Here, we sought to examine metabolites across age in the central nervous system (CNS) (i.e., PFC and HPC) and in the peripheral nervous system (PNS) (i.e., spleen) to gain a better understanding of early alterations in ARCD and AD. The PFC (short-term memory) and HPC (episodic and spatial memory) are two of the first regions affected by AD pathology and two major brain regions proposed to mediate learning and memory processes^27–30^. Recently, the spleen to brain connection has given insight into how splenic inflammation triggers changes in the brain^31^. Thus, making the spleen an important tissue to consider when examining how peripheral metabolites can influence progression of AD pathology. At 6, 12, and 24 months of age, both AD and aged-match Ctrl mice were tested for cognitive impairments in a 3-shock CFC paradigm and the PFC, HPC, and spleen were collected and analyzed for metabolomic alterations. We report that AD mice exhibited memory deficits at 6 months of age and that these memory deficits were also seen in aged mice at 24 months of age. By dissecting and separating both the left and right hemispheres of the PFC and HPC, we found right-lateralized changes in metabolites between Ctrl and AD mice across ages. Lastly, there were common pathways altered in AD and Ctrl mice, but also a divergence in the metabolome between ARCD and AD across ages in different tissues. Since metabolic pathways are largely conserved between species, these results could improve the translation of preclinical research^32^.

## Materials and methods

### Mice

Male ArcCreER^T2^(+) mice were bred with ROSA26-CAG-stop^flox^-channelrhodopsin2 (ChR2)(H134R)-enhanced yellow fluorescent protein (EYFP) (Ai32)^33^ onto a 129S6/SvEv background and were used in all experiments. Triple transgenic lines were then generated^34^ in which male ArcCreER^T2^(+) × ChR2-EYFP homozygous (f/f) mice were bred with a mouse model of AD, female Tg(APPswe; PSEN1dE9) 85Dbo/Mmjax (APP/PS1) mice (034832-JAX, MMRRC)^35^. The Ctrl mice were: APP/PS1(−) × ArcCreER^T2^(+) or (−) × ChR2-EYFP(+/f). The AD mice were: APP/PS1(+) × ArcCreER^T2^(+) or (−) × ChR2-EYFP(+/f). Male mice were used in all experiments. All mice were housed in a 12-h (06:00–18:00) light–dark colony room at 22 °C. Food and water were provided *ad libitum*. All procedures were conducted in accordance with the National Institutes of Health regulations and approved by the Institutional Animal Care and Use Committees (IACUC) of the New York State Psychiatric Institute (NYSPI) under protocol # 1439.

### Genotyping

The Cre and ChR2 genotyping was performed as previously described^36^. APP and PSEN genotyping were performed as described on the Jackson Laboratory website (https://www.jax.org/strain/005864). All genotyping was performed separately.

### Contextual fear conditioning (CFC)

A 3-shock CFC procedure was administered as previously published^37,38^. Briefly, mice were placed into context A and administered 3 2-s shocks (0.75 mA) at 180 s, 240 s, or 300 s following placement into context A. Mice were removed from the context 15 s following the termination of shock (at 317 s). For context retrieval, mice were placed back into context A for 300 s. All sessions were scored for freezing using FreezeView4. The CFC chambers were cleaned with 70% ethanol (EtOH) before and in between trials. Behavioral testing occurred during the light phase and mice underwent testing at 6, 12, or 24 months of age.

A repeated measures analysis of variance was run for training day across time using the JMP statistical package (SAS, Cary, NC, USA) (Table S1). ANOVAs were performed for Time, Genotype, Age, and all interactions. Alpha was set to 0.05 for all analyses. Student *t*-tests were run for all post-hoc behavioral tests.

### Brain extraction

Mice were euthanized via cervical dislocation 2 h following CFC re-exposure. Brains were extracted and flash frozen on dry ice for 30 s. Brains were then cut using a brain matrix slicer (Cat. # BSMAS001-1, Zivic Instruments, Pittsburgh, PA, USA). The PFC and HPC tissues were manually dissected and cut bilaterally. Left and right hemispheres were stored separately. The PFC was cut − 2.0 mm from the olfactory bulbs. After placing the brain into the matrix, we measured − 2.0 mm from the bulbs by counting 2 slice sections (1.0 mm each). Each sample was weighed and then stored at − 80 °C until metabolomic analyses. All brain tissue sample weights are included in Table S2.

### Spleen collection

Spleens were dissected from the abdominal cavity immediately following decapitation. Each sample was weighed and transferred to an Eppendorf tube, which was stored at − 80 °C until metabolomic analyses. All spleen sample weights are included in Table S2.

### Preparation of brain tissue

**Figure 1:**
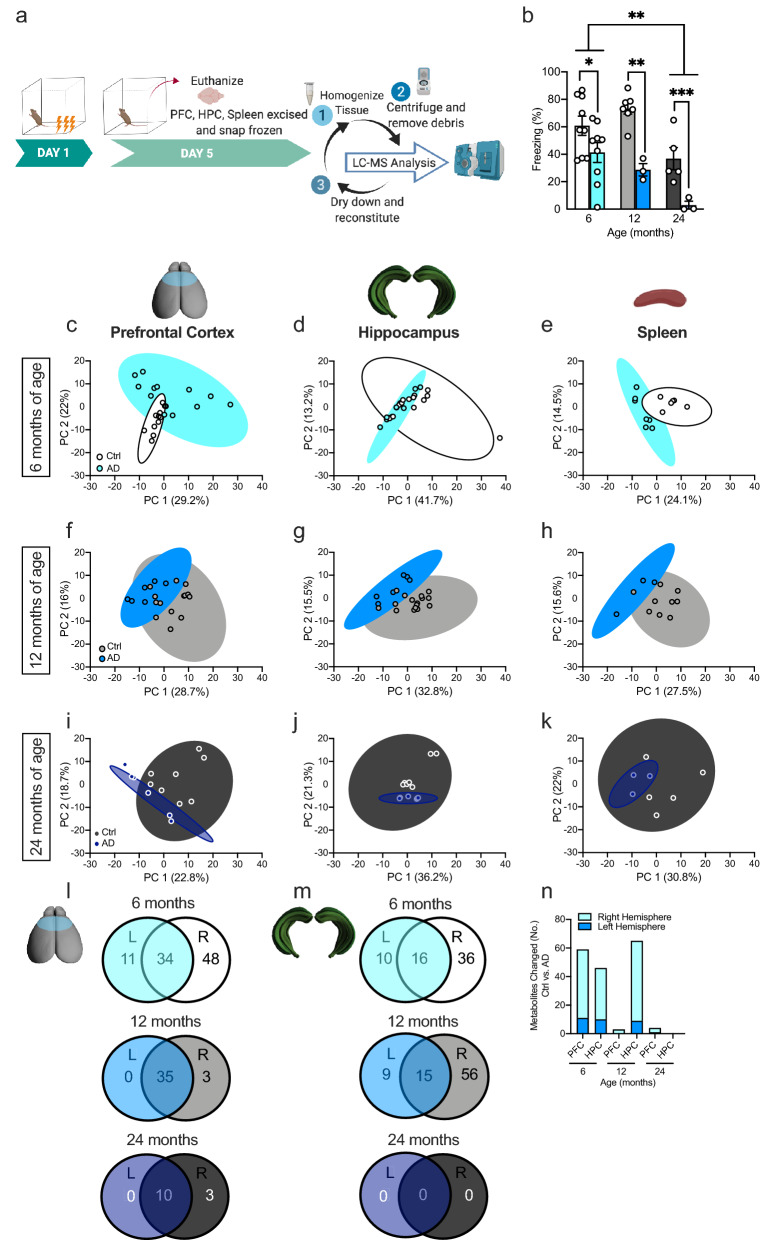
Metabolite profiles between Ctrl and AD mice begin to overlap at 24 months of age. (**a**) Experimental design. (**b**) Following 3-shock CFC training, AD mice exhibited significantly less freezing behavior when compared to Ctrl mice at 6, 12, and 24 months of age. Twenty-four-month-old Ctrl mice exhibited significantly less freezing than 6-month-old Ctrl mice. Metabolites of both Ctrl and AD mice were assessed in the prefrontal cortex, hippocampus, and spleen of (**c**–**e**) 6-month-old, (**f**–**h**) 12-month-old, and (**i**–**k**) 24-month-old mice. (**l**–**m)** The Venn diagrams show the overlap between metabolites changed in both the left and right hemispheres. (**n**) The bar graph displays the number of changed metabolites in each hemisphere, per region, across age. (n = 3–6 mice per group). Error bars represent ± SEM. *p < 0.05; **p < 0.01; ***p < 0.001. *Ctrl* control, *AD* Alzheimer’s disease, *PFC* prefrontal cortex, *HPC* hippocampus, *LC–MS* liquid chromatography–mass spectrometry.

Frozen brain tissue was transferred to a homogenization tube containing ceramic beads as previously described in McGowen et al.^39^. Briefly, ice-cold 80% MeOH/20% H_2_O was added to obtain a 20 mg/ml solution to each of the tubes. The tubes were snap frozen in liquid nitrogen during processing. After a brief thawing period, the tubes were then transferred to a Bead Ruptor Homogenizer (Omni International, Kennesaw, GA, USA). Tissue was homogenized, sonicated, and then centrifuged (Fig. 1a). All of the supernatant was removed, transferred to an Eppendorf tube, and evaporated to dryness overnight using a centrifugal evaporator. Once dry, the dried lysate was stored at − 80 °C until analysis.

### Preparation of spleen tissue

The spleen tissue was homogenized using the same procedure as the brain tissue. After the samples were pulverized, sonicated, and centrifuged the supernatant from each sample was transferred into two Eppendorf tubes, 400 μl into each tube. Both sets of sample aliquots were dried overnight on a DNA 120 Speedvac (ThermoFisher, Waltham, MA, USA). Once dry, the lysate was stored at − 80 °C until analysis.

### Metabolomics analysis of brain tissue

Brain samples were analyzed using an untargeted metabolomics platform as previously described in McGowan et al.^39^. Briefly, all samples were reconstituted in 2:1:1 acetonitrile:MeOH:H_2_O, to yield a concentration of 200 mg/ml, followed by centrifugation at 14,000 rpm for 10 min at 4 °C to remove excess debris before analysis. Chromatography was performed using an Agilent 1290 Infinity UPLC (Agilent, Santa Clara, CA, USA). 10 μl of each sample were injected onto a ZIC-pHILIC column (EMD Millipore, Billerica, MA, USA) with dimensions of 150 × 4.6 mm, 5 μm. Metabolites were separated using an acetonitrile/H_2_O with 20 mM ammonium carbonate (pH 9.2) gradient over a 29-min period. A 10-min re-equilibration time was carried out in between injections. Detection was performed using an Agilent 6550 quadrupole-time-of-flight (QToF) mass spectrometer (Agilent, Santa Clara, CA, USA), operated in both negative and positive modes. Full scan MS data was collected from 70–1,000 m/z and metabolites were identified in an untargeted manner by looking within 10 ppm of the expected m/z values. Real-time mass calibration was performed throughout the duration of sample analysis.

### Metabolomics analysis of spleen tissue

In order to expand metabolite coverage, the spleen tissue samples were reconstituted and analyzed using 3 instrumental methods: targeted MRM HILIC–LC–MS/MS analysis, untargeted HILIC–LC–QTOF analysis, and untargeted GC–MS analysis. One of the two sets of dried spleen samples was reconstituted with a calculated volume of cold 3:3:2 IPA:ACN:H_2_O to bring the final concentration of each sample to 0.075 mg tissue/µl. After reconstitution, 100 µl of supernatant was aliquoted into vials for targeted MRM HILIC–LC–MS/MS analysis on a Shimadzu NEXERA UPLC (Shimadzu, Columbia, MD, USA) connected to a SciEx 5500 Triple-Quadropole mass spectrometer (SciEx, Framingham, MA, USA). A further 70 µl was aliquoted into Eppendorf tubes and again dried overnight on a DNA 120 Speedvac (ThermoFisher, Waltham, MA, USA). The 70 µl dried aliquots were then derivatized and diluted with n-hexanes for untargeted GC–MS analysis. GC–MS analysis was done using an Agilent 7890B gas chromatograph (Agilent, Santa Clara, CA, USA) interfaced to a time-of-flight (TOF) Pegasus HT mass spectrometer (LECO, St. Joseph, MI, USA) with automated injections using an MPS2 programmable robotic multipurpose sampler (Gerstel, Muhlheim an der Ruhr, Germany). All derivatization, HILIC–LC–MS/MS, and GC–MS analyses were performed as previously outlined in Drolet et al.^40^. The second set of dried spleen sample aliquots were reconstituted into a calculated volume of 100% Optima Water to bring the final concentration of each sample to 0.2 mg tissue/µl. After reconstitution the samples were subjected to analysis of metabolites involved with neurotransmission on an Agilent 6550 quadrupole-time-of-flight (QToF) mass spectrometer (Agilent, Santa Clara, CA, USA) as outlined in the analysis performed in McGowen et al.^39^.

### MetaboAnalyst analysis

#### Data normalization

Raw peak intensity values for each metabolite were uploaded to MetaboAnalyst for each tissue sample (i.e., PFC, HPC, and spleen). Data was normalized by the median with a log transformation. Auto scaling was also performed. The normalized data for each tissue was then used for statistical analysis (reported as, *normalized peak area*).

#### Principal component analysis (PCA)

Normalized data at each age was entered into MetaboAnalyst and run through PCA. The data from the 2D scores plot of Ctrl vs. AD mice were then exported and transferred to Prism8 (Graphpad, San Diego, CA, USA) to create the graphical representation. An across age PCA was run by entering the data from the normalized Ctrl group separately from the normalized AD group into MetaboAnalyst.

#### ANOVA

Normalized data at each age were entered into MetaboAnalyst and an ANOVA was performed to determine which metabolites were changed in both the left and right hemispheres for PFC and HPC in Ctrl vs. AD mice. Fisher’s LSD were performed for all post hoc tests. A *t*-test between Ctrl and AD was performed for each metabolite in the spleen. Only the changed metabolites between Ctrl and AD from each age were then graphed across age and ANOVAs were run to determine differences across ages. Metabolite values from each hemisphere were combined. A Tukey HSD was performed for all post hoc tests. Alpha was set to 0.05 for all analyses. Data are expressed as means ± SEM. All statistical tests are included in Tables S3, S4, S5, S6, S7, and S8.

#### Pathway analysis

For each across age pathway analysis, the top 50 metabolites that were changed across age were entered into MetaboAnalyst 3.0 using their HMDB IDs (https://www.metaboanalyst.ca/MetaboAnalyst/faces/upload/PathUploadView.xhtml). For age-specific pathway analysis, all metabolites changed between Ctrl and AD in both hemispheres were entered into MetaboAnalyst 3.0 using their HMDB IDs. Pathway results for PFC and HPC are included in Table S6 and S7.

#### Correlation analysis

Normalized data across age were entered into MetaboAnalyst for Ctrl and AD separately and a correlation analysis was performed against a feature of interest (freezing behavior). The top 50 (25 positive, 25 negative) correlated metabolites were exported and graphed using Prism GraphPad. Using the MetaboAnalyst enrichment analysis, we entered in the correlated metabolites and compared these metabolites to a human library of urine sample metabolites (https://www.metaboanalyst.ca/MetaboAnalyst/faces/upload/EnrichUploadView.xhtml). The stars on the graph indicate metabolites found also in AD human urine samples^41^.

## Results

### ARCD and AD mice exhibit contextual fear learning deficits

We and others have previously reported that AD (APP/PS1) mice exhibit memory deficits starting at 6 months of age^34,42^. However, it remained to be determined if any metabolomic changes correlated with these cognitive deficits. Here, we tested Ctrl and AD mice at 3 separate ages in a 3-shock CFC paradigm to assay memory retrieval (Fig. 1b). Mice were administered a 3-shock CFC paradigm and 5 days later, were administered a context re-exposure. Freezing behavior, time when the mouse is still, was used as measure of memory impairment. As previously reported, AD mice exhibited less freezing behavior at 6 months of age when compared to Ctrl mice. This difference persisted when separate groups of mice were tested at 12 and 24 months of age. In addition, we showed ARCD from 6 to 24 months of age in Ctrl mice (Fig. 1b). These data indicate that there are robust memory retrieval deficits in fear memory retrieval in ARCD and AD mice.

### Metabolite profiles between Ctrl and AD mice begin to overlap at 24 months of age

Following CFC, mice were euthanized, brain tissue and spleen were collected, and metabolomic analyses were performed (Fig. 1a). AD mice exhibited cognitive decline at 6, 12, and 24 months of age compared to Ctrls. ARCD was also observed in naturally aged mice at 24 months of age (Fig. 1b). Through the use of a combined untargeted/targeted metabolomic analysis approach, 323 metabolites were altered across ages in the PFC, 216 in the HPC, and 254 in the spleen. These changes occurred in both the left and right hemispheres in the PFC and HPC. Using PCA, the metabolite profiles separated Ctrl from AD mice at 6 and 12 months of age in the PFC, HPC, and spleen (Fig. 1c–h). Metabolite profiles in the PFC continued to show separation between the groups at 24 months of age (Fig. 1i). However, in the HPC and spleen at 24 months of age, metabolite profiles were indistinguishable between Ctrl and AD mice (Fig. 1j,k). This suggests that natural aging results in similar metabolic pathway changes as AD by 24 months of age. This is also observed behaviorally at 24 months of age, as Ctrl and AD mice exhibit similar levels of cognitive decline. The total number of metabolites that showed a statistically significant change between Ctrl and AD were 79 in the PFC, 31 in the HPC, and 18 in the spleen (Fig. 1l–n).

### Right-lateralized changes in metabolites in the PFC and HPC

Lateralized changes in the brain have become a recent area of interest in the AD field^43^. For example, when examining functional lateralization in humans, abnormal rightward dominance is observed in patients with MCI and AD^43^. Metabolite differences between Ctrl and AD mice were then analyzed separately in each hemisphere of the PFC and HPC. The number of significantly changed metabolites between Ctrl and AD mice are shown in the Venn diagrams, with the overlap representing the number of changed metabolites in both hemispheres (Fig. 1l,m). Most notably, in the PFC and HPC at 6 months of age there were more metabolomic changes in the right hemispheres when compared to the left hemispheres (Fig. 1n). At 12 months of age, this difference persisted only in the HPC and by 24 months of age, there was little difference between the number of metabolites changed in left versus right hemispheres. These data indicate that the right hemisphere is attempting to compensate for the degeneration occurring throughout the brain, but with age, both hemispheres become impaired.

### Metabolites negatively correlated with freezing behavior are also observed in human AD samples

**Figure 2:**
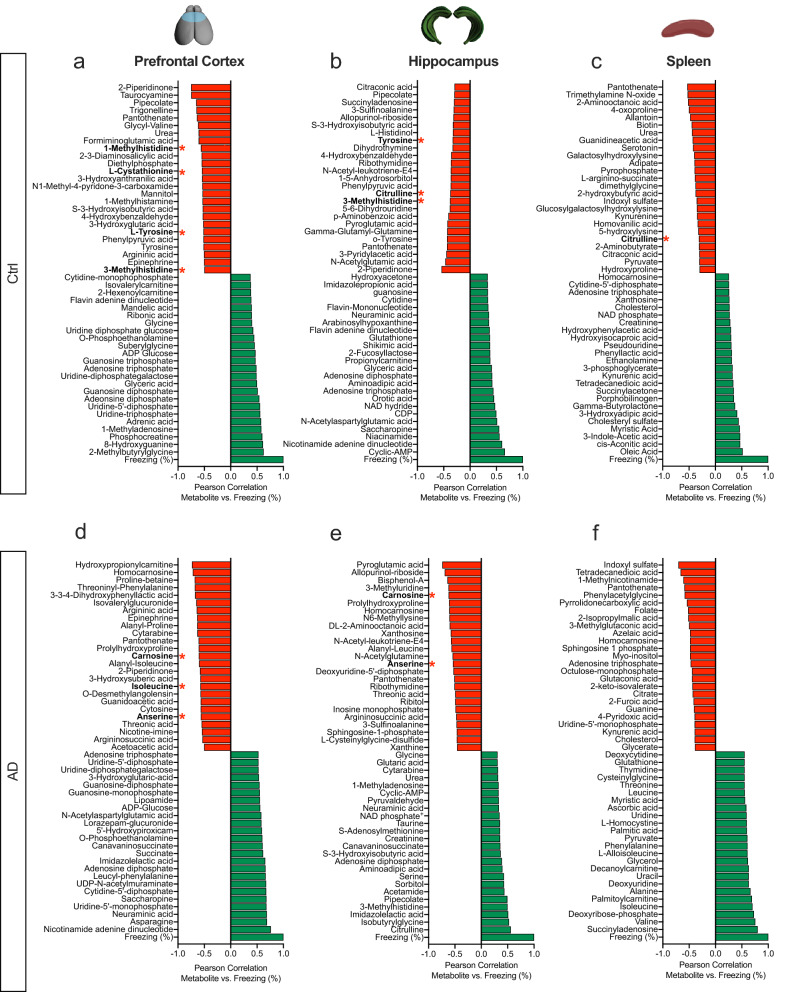
Metabolites negatively correlated with freezing behavior are also observed in human AD urine samples. (**a**–**c**) Graphs representing the top metabolites positively and negatively correlated with freezing behavior across ages in the PFC, HPC, and spleen of Ctrl mice. (**d**–**f**) Graphs representing the top metabolites positively and negatively correlated with freezing behavior across ages in the PFC, HPC, and spleen of AD mice. The red bars denote negative correlations and the green bars denote positive correlations to freezing behavior. The red stars indicate metabolites that are changed in AD human urine samples when compared to a library using MetaboAnalyst. *Ctrl* control, *AD* Alzheimer’s disease, *PFC* prefrontal cortex, *HPC* hippocampus.

To determine if there was a correlation between metabolites and memory impairment, we next ran correlation analyses between metabolites and freezing behavior (%) across ages within groups for each tissue type. The top 25 negative (red) and positive (green) correlations were graphed for each tissue sample across age (Fig. 2a–f). Pathway analysis revealed purine and pyrimidine metabolic pathways were positively correlated with freezing behavior in the PFC, HPC, and spleen of Ctrl and AD mice. However, there were no common pathways between tissues when examining the negatively correlated metabolites. Therefore, an enrichment pathway analysis was run using MetaboAnalyst. This module performs metabolite set enrichment analysis (MSEA) for human and mammalian species based on several libraries containing ~ 6,300 groups of metabolite sets^44,45^. The MSEA has libraries from blood, urine, and CSF metabolite sets.

After comparing the three libraries, human urine samples were most related to our dataset. Ultimately, this would provide the least invasive test for detecting biomarkers. In both Ctrl and AD mice, metabolites with negative correlations to freezing behavior were also found in human AD urine samples (red asterisks). These metabolites were found in all three tissue samples except for the spleen of AD mice. Correlation analyses were run between the metabolites that aligned with the human AD sample database. Significant negative correlations for each metabolite in the PFC and HPC were observed (Supplemental Fig. S1). In Ctrl mice, 1-methylhistidine, l-cystathionine, l-tyrosine, and 3-methylhistidine levels in the PFC were negatively correlated with freezing behavior across age (Supplemental Fig. S1a–d). 3-methylhistidine and tyrosine were also significant in the HPC (Supplemental Fig. S1e,f). Interestingly, Citrulline was common in the HPC and spleen, representing a connection between the PNS and CNS as negative correlations between metabolite and behavior were observed (Supplemental Fig. S1g,h). However, the correlation to freezing behavior was not significant in the spleen suggesting that the PNS may not directly influence cognitive decline. In AD mice, carnosine, anserine, and isoleucine were negatively correlated with freezing behavior in both the PFC and HPC (Supplemental Fig. S1i–k). Anserine and carnosine were also observed in the HPC (Supplemental Fig. S1l,m). All of the negatively correlated metabolites are also known as free amino acids which are involved in histidine and dopamine metabolism^41^. These data suggest that specific metabolites across ages can impact cognitive decline and that our data is translatable to humans.

### AD impacts histidine metabolism in the PFC, HPC, and spleen

**Figure 3:**
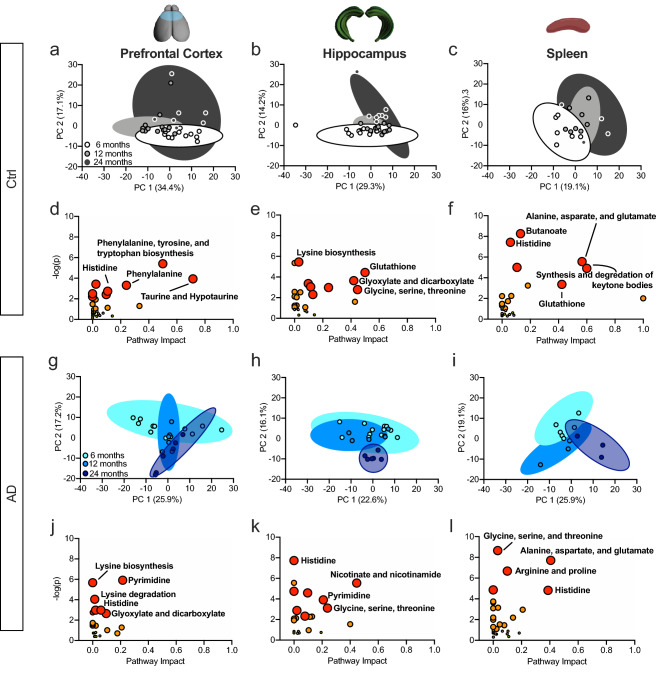
AD mice exhibit altered histidine metabolism in the prefrontal cortex, hippocampus, and spleen. (**a**–**c**) PCA revealed a separation across age in Ctrl metabolite profiles in the PFC, HPC, and spleen. (**d**–**f**) Pathway analysis of the metabolic pathways most affected throughout aging in the PFC, HPC, and spleen. Histidine metabolism was altered in the PFC and spleen while glutathione was altered in the HPC and spleen. (**g**–**i**) PCA revealed a separation across age in AD metabolite profiles in the PFC, HPC, and spleen. (**j**–**l**) Pathway analysis of the metabolic pathways most affected in AD in the PFC, HPC, and spleen. Histidine metabolism was altered in AD mice across tissue samples.

Because metabolite changes across ages within groups were observed, a PCA was run to determine the effect of natural aging on metabolite profiles. The PCA revealed a separation within groups across ages in each tissue sample in Ctrl mice using the metabolite concentrations (Fig. 3a–c). A pathway analysis of changed metabolites across age revealed Ctrl mice did not display a common pathway in every tissue (Fig. 3d–f), but did show changes in histidine in the PFC and Spleen (Fig. 3d,f). Ctrl mice also exhibited changes in the glutathione pathway in the HPC and spleen (Fig. 3e,f).

In AD mice, the PCA also revealed a separation within groups across ages in each tissue sample (Fig. 3g–i). The pathway analysis of changed metabolites across age revealed that histidine metabolism was affected in all tissue samples (Fig. 3j–l). All metabolites related to the histidine pathway are graphed in Supplemental Fig. S2. AD mice also exhibited changes in the pyrimidine pathway in the PFC and HPC (Fig. 3j,k). The glycine, serine, and threonine pathway was altered in the HPC and spleen of AD mice (Fig. 3k,l).

Although Ctrl and AD mice show divergence in the metabolome across age, there are also similar pathways affected. For example, in the PFC, Ctrl and AD mice show alterations in histidine metabolism (Fig. 3d,j), in the HPC changes are observed in glycine, serine, and threonine pathways (Fig. 3e,k), and in the spleen similarities are seen in the alanine, aspartate, and glutamate pathways (Fig. 3f,l). In summary, metabolic profiles were separated by age in both Ctrl and AD mice. Histidine was a common pathway impacted by aging in AD mice.

### AD significantly alters amino acid catabolism and energy metabolism pathways in the PFC

Metabolites significantly changed in both hemispheres between Ctrl and AD mice at each age were used in a pathway analysis to determine the most significant metabolic pathways. Four pathways were chosen in the PFC as significant in terms of *p* value and impact score (Table S6).

**Figure 4:**
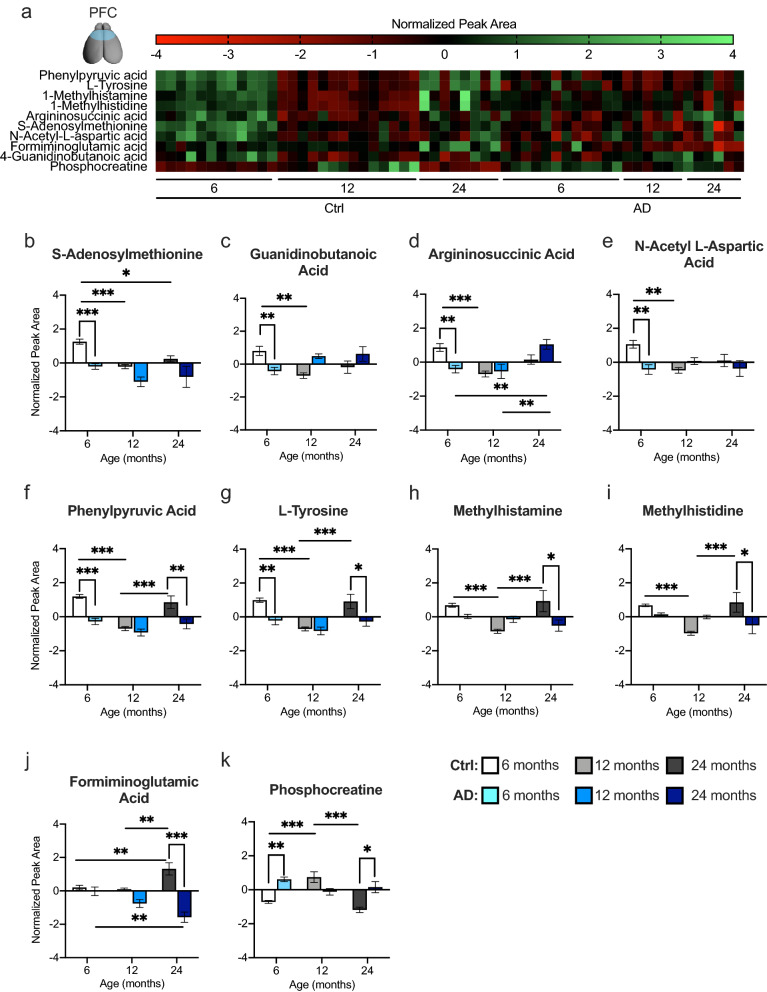
AD mice exhibit altered amino acid catabolism and energy metabolism pathways in the prefrontal cortex. (**a**) A heat map expressing the normalized peak area concentrations of the top 10 metabolites that are changed between Ctrl and AD mice across age in the PFC. Green indicates greater concentration and red indicates weaker concentration. (**b**–**k**) Top 10 metabolites significantly upregulated or downregulated between Ctrl and AD mice in the PFC at the three ages tested. These metabolites are in the phenylalanine, tyrosine and tryptophan (phenylpyruvic acid, l-tyrosine, 1-methylhistamine, 1-methylhistidine); arginine and proline (argininosuccinic acid, s-adenosylmethionine, phosphocreatine, 4-guanidinobutanoic acid); alanine, aspartate, and glutamate (n-acetyl-l-aspartic acid, argininosuccinic acid), and histidine (1-methylhistamine, formiminoglutamic acid, 1-methylhistidine) metabolic pathways. (n = 3–6 male mice per group). Error bars represent ± SEM. *p < 0.05; **p < 0.01; ***p < 0.001. *Ctrl* control, *AD* Alzheimer’s disease.

In the PFC, 10 metabolites were changed in phenylalanine, tyrosine, and tryptophan biosynthesis, histidine metabolism, and arginine, proline, alanine, aspartate, and glutamate metabolism pathways (Fig. 4). The heat map displays the top 10 metabolite normalized concentrations across age for Ctrl and AD mice (Fig. 4a). At 6 months of age, AD mice exhibited significantly lower concentrations in 6 out of the 10 metabolites when compared to Ctrl mice (Fig. 4b–g). With age, many of these metabolites decreased in Ctrl mice. Specifically, at 12 months of age, Ctrl and AD mice exhibited similar concentrations of all 10 metabolites. However, by 24 months of age, AD mice displayed lower concentrations of phenylpyruvic acid (Fig. 4f), l-tyrosine (Fig. 4g), methylhistamine (Fig. 4h), methylhistidine (Fig. 4i), and formiminoglutamic acid (Fig. 4j) when compared to Ctrl mice. Interestingly, phosphocreatine, was the only metabolite significantly increased in AD mice at six and 24 months of age (Fig. 4k). These results suggest that multiple metabolites relating to amino acid catabolism and energy metabolism may be informative biomarkers at different ages. The first 6 metabolites that were decreased at 6 months of age in AD mice are more relevant for early biomarker detection.

### AD significantly alters protein synthesis and oxidative stress in the HPC

**Figure 5:**
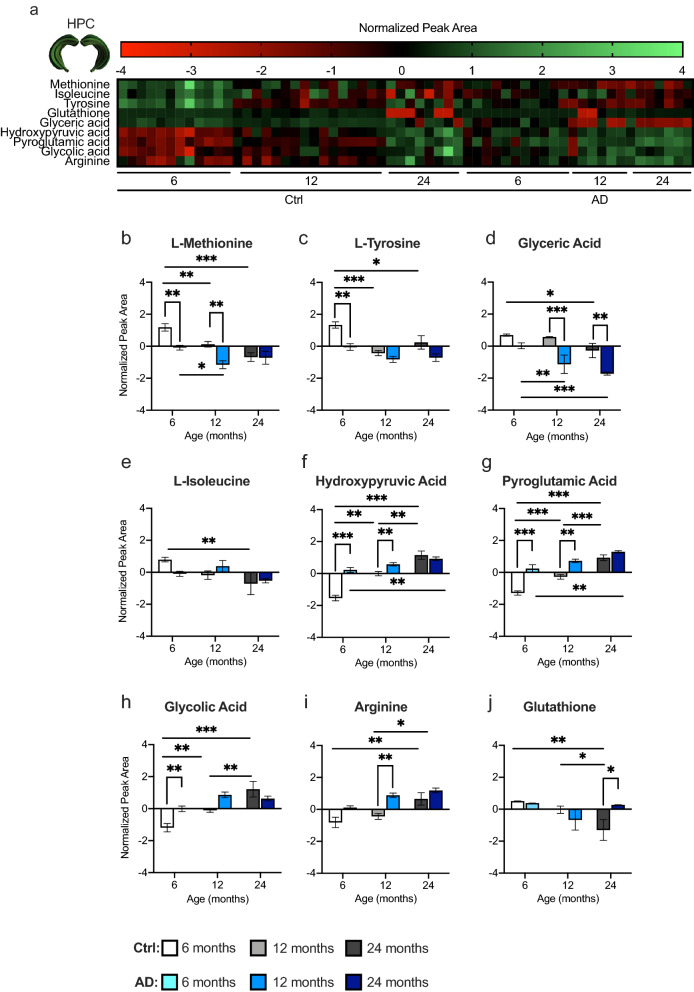
AD mice exhibit altered protein synthesis and oxidative stress metabolic pathways in the hippocampus. (**a**) A heat map expressing the normalized peak area concentrations of the top 9 metabolites that are changed between Ctrl and AD mice across age in the HPC. Green indicates greater concentration and red indicates weaker concentration. (**b**–**j**) Top 9 metabolites significantly upregulated or downregulated between Ctrl and AD mice in the HPC at different ages. These metabolites are in the aminoacyl-tRNA biosynthesis (l-methionine, isoleucine, tyrosine, arginine); glutathione (glutathione, pyroglutamic acid); and glyoxylate and dicarboxylate (hydroxypyruvic acid, glycolic acid, glyceric acid) metabolic pathways. (n = 3–6 male mice per group). Error bars represent ± SEM. *p < 0.05; **p < 0.01; ***p < 0.001. *Ctrl* control, *AD* Alzheimer’s disease.

Metabolites significantly changed in both hemispheres between Ctrl and AD mice at each age were used in a pathway analysis to determine the most significant metabolic pathways in the HPC. The heat map displays the top 9 HPC metabolite normalized concentrations across age for Ctrl and AD mice (Fig. 5a). Three pathways were chosen as significant in terms of p value and impact score (Table S7). Data from the HPC revealed changes in aminoacyl-tRNA biosynthesis, glutathione, and glyoxylate and dicarboxylate metabolic pathways. Of the 9 metabolites changed within these pathways, 2 were significantly decreased in AD mice when compared to Ctrl mice at 6 months of age (Fig. 5b,c). By 12 and 24 months of age, AD mice began to show a decrease in glyceric acid levels compared to Ctrl mice (Fig. 5d). l-isoleucine levels were similar across ages between Ctrl and AD mice; however, this metabolite was specific to ARCD as Ctrl mice showed a decrease in levels from 6 to 24 months of age (Fig. 5e).

Three metabolites were significantly increased in the HPC of AD mice when compared with Ctrl mice (Fig. 5f–h). Hydroxypyruvic acid (Fig. 5f), pyroglutamic acid (Fig. 5g), and glycolic acid (Fig. 5h) were increased at 6 months of age, while arginine was increased at 12 months (Fig. 5i) in AD mice. However, by 24 months of age all 4 metabolites were similar between Ctrl and AD mice. Interestingly, hydroxypyruvic and pyroglutamic acid increased across ages in both Ctrl and AD mice, while glycolic acid and arginine increased only in Ctrl mice across age. Glutathione was the only metabolite that was increased in AD mice at 24 months of age relative to Ctrl mice (Fig. 5j). This metabolite decreased across ages in Ctrl mice, but did not change across age in AD mice, suggesting that glutathione is specifically related to ARCD^46^. In addition, all of the changed metabolites are implicated in protein synthesis and oxidative stress, both of which have been implicated in AD pathology^47,48^.

### Age-related metabolite changes occur earlier in the spleen compared to the CNS

**Figure 6:**
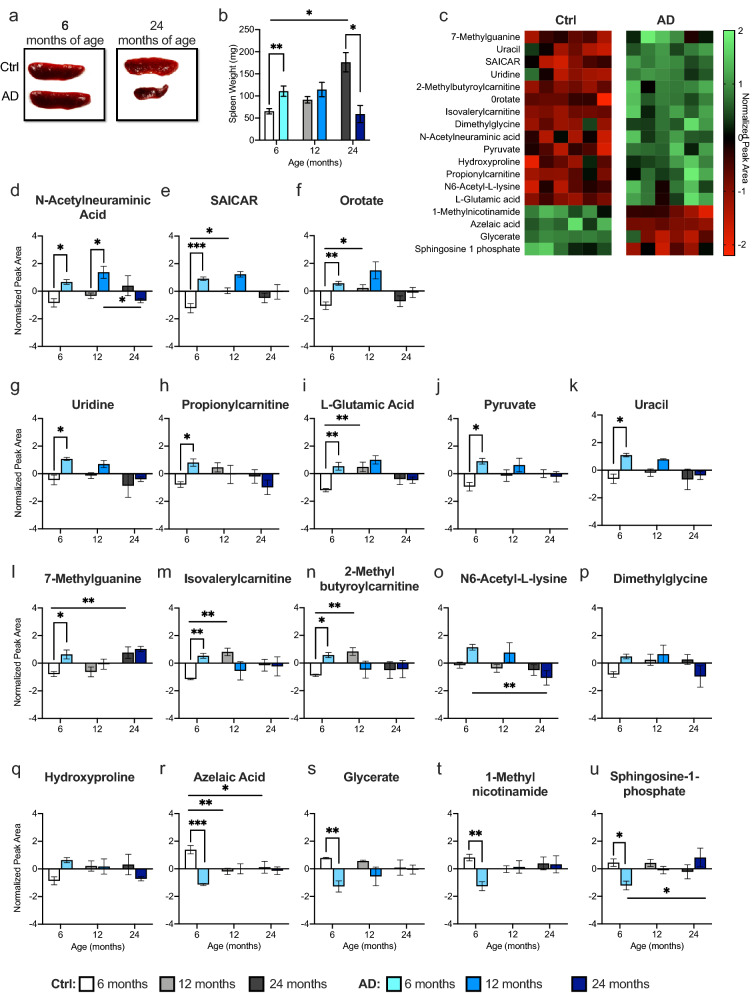
Splenomegaly contributes to early metabolite changes in AD mice at 6 months of age. (**a**) Representative images of spleens from 6- and 24-month-old Ctrl and AD mice. (**b**) AD mice exhibited significantly enlarged spleens at 6 months of age when compared to Ctrl mice. However, at 24 months of age, AD mice had significantly smaller spleens when compared with Ctrl mice. (**c**) Heat map of the 18 changed metabolites between Ctrl and AD mice at 6 months of age. Green indicates greater concentration and red indicates weaker concentration. (**d**–**n**) AD mice exhibited an increase in 11 out of the 18 metabolites altered in the spleen at 6 months of age. Ctrl and AD mice had similar metabolite levels at 12 and 24 months of age. (**o**–**q**) In 3 out of 18 metabolites, Ctrl and AD mice displayed similar levels of metabolites at each age. (**r**–**u**) AD mice showed decreased metabolite levels in 4 out of 18 metabolites (n = 3–6 male mice per group). Error bars represent ± SEM. *p < 0.05; **p < 0.01; ***p < 0.001. *Ctrl* control, *AD* Alzheimer’s disease, *mg* milligram, *SAICAR* phosphoribosylaminoimidazolesuccinocarboxamide.

Because the spleen is connected to the CNS through the splenic and vagus nerve and communicates with the brain^31,49,50^, we next ran metabolomic analysis on spleen tissue to determine if metabolite alterations in the periphery contributed to the cognitive decline we observed following ARCD and AD. First, we observed that the spleens were significantly enlarged in AD mice when compared to Ctrl mice at 6 months of age (Fig. 6a,b). At 12 months of age, Ctrl and AD mice exhibited a comparable spleen weight. However, at 24 months of age, AD mice had significantly smaller spleens when compared with Ctrl mice. The spleen became significantly enlarged with age in Ctrl mice.

At 6 months of age, 18 metabolites were significantly different between Ctrl and AD mice (Fig. 6c). N-acetylneuraminic acid was the only metabolite significantly different between Ctrl and AD mice at 12 months of age (Fig. 6d). Ctrl and AD mice exhibited similar metabolite levels at 24 months of age in all 18 metabolites. Pathway analysis revealed that the changed metabolites are part of the glycine, serine, threonine, and pyrimidine metabolic pathways. At 6 months of age, AD mice exhibited a significant increase in 11 out of the 18 metabolites (Fig. 6d–n). Significant interactions (Group × Age) were observed for N6-acetyl-lysine, dimethylglycine, and hydroxyproline, but post hoc tests did not reveal any significant differences (Fig. 6o–q). However, 4 metabolites were decreased in AD mice when compared with Ctrl mice at 6 months of age (Fig. 6r–u). These data suggest that the periphery exhibited changes at an earlier age and that by 12 and 24 months of age it is difficult to distinguish between Ctrl and AD mice. It may be beneficial to examine the periphery at an earlier age to discover potential biomarkers.

## Discussion

Here, we sought to identify metabolomic changes following ARCD and AD that could begin to elucidate cognitive decline resulting from both processes. We report that while AD mice exhibit a memory retrieval impairment at 6 months of age, aged mice do not exhibit a memory retrieval impairment until 24 months of age. This decline in memory was associated with a robust change in metabolite profiles between AD and ARCD. Interestingly, this change was most prominent in the right hemisphere in both the PFC and the HPC, suggesting that the brain is compensating for damage in the left hemisphere as seen in human MCI and AD patients^43^. In addition to the CNS, we also examined changes in the spleen. Notably, changes in the spleen were only observed at 6 months of age and may represent an area for early biomarker detection. To our knowledge, this is the first study that identifies metabolite alterations across ages in both aged and AD cohorts in order to identify novel biomarkers for early disease diagnosis.

Potential biomarkers include those that are also correlated with cognitive decline. The correlation analysis across age revealed that negatively correlated metabolites to freezing behavior in mice were also found in the urine of human AD patients^41^. These metabolites are part of the histidine and dopamine pathways, both of which are altered in AD^51–53^. In AD patient cerebral spinal fluid (CSF), histidine was identified as a possible disease progression biomarker^53^. One potential therapeutic strategy for AD patients is to supplement low protein diets with high levels of branched-chain amino acids, such as histidine, glutamine, and threonine^54^.

Dopamine, a neurotransmitter involved in regulating emotional responses, which also plays a role in synaptic plasticity, is reduced in AD patients^55,56^ as well as AD mouse models^51^. Dopamine can be derived from tyrosine, which is negatively correlated to memory in our dataset. Tyrosine increases with age, potentially leading to an increase in 3,4-dihydroxyphenylalanine (l-DOPA) and a decrease in dopamine. Concurrent with our research, tyrosine and l-DOPA concentration is significantly increased in plasma from AD patients while dopamine is reduced^41^. The mechanism for this decrease in dopamine is unclear, but researchers suspect this is due to a decrease in decarboxylase activity or decreased conversion from l-DOPA to dopamine, in AD patients.

When looking across ages, metabolite profiles were able to separate ages in each tissue within each group. The glutathione pathway was altered specifically in Ctrl mice across ages in both the spleen and HPC. Glutathione, an antioxidant present in almost every cell in the body, is decreased during aging and could represent a therapeutic target to slow the aging process^57,58^. Pyrimidine metabolic pathways, typically known as precursors for nucleic acid synthesis, were specific to the CNS in AD mice. Pyrimidine also plays a role in phospholipid biosynthesis, detoxification processes, and protein lipid glycosylation^59,60^. The decrease in pyrimidine synthesis of nucleotides may contribute to the dysfunction of oxidative phosphorylation (OXPHOS) which then leads to the pathogenesis of late onset AD through impaired cellular respiration^61,62^.

When examining each tissue separately, multiple changes in metabolic pathways related to energy metabolism were seen in the PFC. In addition to energy metabolism, there was also an impact in the tryptophan, phenylalanine, and tyrosine pathways. All of these amino acids are part of the central synthesis of neurotransmitters, serotonin, dopamine, and norepinephrine^63^. Overall, AD mice exhibited a decrease in the phenylpyruvic acid, l-tyrosine, methylhistamine, and methylhistidine metabolites. This decrease is also seen in previous metabolic reports examining serum from AD patients^64^. However, others have reported an increase in both phenylalanine and tryptophan in the brains of AD patients^65^. The mechanisms for these changes remain unclear, but as a class, aromatic amino acid metabolism is essential for neuronal functioning. In fact, argininosuccinic acid, a precursor of arginine upregulated across age in our AD mice, can lead to increased synthesis of guanidinobutanoic acid, which is capable of impairing the nervous system^66^. This is because arginine acts on the nitric oxide (NO) pathway^67^, which can lead to neurotoxicity and neurodegeneration^68–71^. Interestingly, phosphocreatine was the only metabolite increased in AD mice at 6 months of age. Phosphocreatine is essential for energy production and balance however, an increase in levels could indicate a reduction in the utilization of ATP^72^. These results corroborate with human studies showing that phosphocreatine is also increased in post-mortem hippocampal human AD patient samples^73^. This increase specifically occurred in regions that show early degeneration in AD indicating an altered energy metabolism in mild AD. Phosphocreatine levels were similar between Ctrls and AD mice at 12 months, suggesting that phosphocreatine increases with normal aging as seen in non-demented older adults^72^. Formiminoglutamic acid, which was decreased in AD mice, is an intermediate in the catabolism of l-histidine to l-glutamic acid and is a marker of folate levels. Many studies have reported that folate levels are lower in AD patients compared to normal Ctrls^74^. In addition, folic acid supplements have been proposed as possible treatments for AD or to reduce AD risk^75^.

We also observed deficits in energy production in AD mice at an early age in the HPC, but alterations in aminoacyl-tRNA (AAR) biosynthesis were specific to the HPC. AAR synthetases are enzymes that join amino acids to tRNAs^76^. They are essential for protein synthesis, transcription, translation, angiogenesis, and apoptosis. AAR biosynthesis is also a common pathway affected in the plasma of AD and MCI patients^21^. Out of the four metabolites belonging to the AAR pathway, 3 were decreased in AD mice. Arginine, however, was increased at 6 and 12 months of age in AD mice compared to Ctrl mice. This increase in arginine could be explained by the increase in argininosuccinic acid observed in the PFC, which leads to neurotoxicity. However, the exact mechanisms associated with AAR and AD are unknown and warrant further investigation. Glutathione and glyoxylate pathways were also altered in the HPC. As mentioned previously, glutathione is decreased with age. However, pyroglutamic acid is also part of the glutathione pathway and is increased in AD mice. This natural amino acid derivative can be converted to glutamate, but surprisingly also plays a role in amyloid production. In 1992, Mori et al. discovered that 15–20% of total Aβ contained a pyroglutamate residue at the N terminus (Aβ_pE3_)^77^. Since its discovery many researchers have identified Aβ_pE3_ as a more aggregated form of Aβ with enhanced β-sheet formation^78^.

Recently, scientists became aware of the spleen to brain connections, which are important for immune responses. Specifically, the splenic nerve connects to the vagus nerve which is connected to brain stem^79, 80^. For example, if acetylcholine receptors on the spleen are stimulated, proinflammatory cytokines are inhibited, which has been shown to improve outcomes in animal models of stroke and traumatic brain injury^81,82^. Splenomegaly, enlargement of the spleen, was observed in the AD mice at 6 months of age, but with age, the spleen size increased in Ctrl mice and was larger than the spleen size in AD mice at 24 months of age. Splenomegaly has also been seen in the 3xTg-AD mouse model. In this model, larger spleens were seen at 24 months of age and were associated with altered cytokine levels in plasm^83^. An enlarged spleen can be caused by infections and other diseases^84^, which may explain the increase in size of the AD spleens at 6 months of age and later the increase in Ctrl spleen size at 24 months of age. However, more information is needed to understand why and how this occurs. In the spleen, glycine, serine, and threonine, and pyrimidine pathways were most affected. These same pathways were altered in the HPC of Ctrl and AD mice suggesting a brain-to-spleen connection. In addition, human studies report that glycine, serine, and threonine metabolism are one of the six metabolic pathways that distinguish cognitively normal Ctrls from AD patients^85^. Because changes in the spleen were only observed at 6 months, it may be beneficial to examine the periphery at an earlier age to discover potential biomarkers.

In summary, taking a metabolomic approach across age has allowed for the identification of pathways that impact Ctrl and AD mice at each age as well as pathways that alter ARCD. We’ve identified that histidine and dopamine metabolism negatively correlate with cognitive decline in all three tissue samples. Furthermore, energy metabolism and protein synthesis pathways were altered in the PFC and HPC. There is also evidence that metabolic changes in the periphery occur earlier in AD mice as changes in the spleen were only observed at 6 months and levels became similar between Ctrl and AD at 12 and 24 months. Future studies will examine metabolic changes at younger ages to determine whether these metabolites can be used to predict disease onset. We can then inhibit or disrupt these metabolite processes to determine how they directly affect disease progression. Additionally, future research will need to address sex differences in the metabolome as females are more susceptible to AD, but the underlying mechanisms for this are not well understood.

## Supplementary information

Supplementary Information 1.
