# Author Correction: Divergence in the metabolome between natural aging and Alzheimer’s disease

**DOI:** 10.1038/s41598-020-75425-7

**Published:** 2020-11-10

**Authors:** Holly C. Hunsberger, Bennett P. Greenwood, Vladimir Tolstikov, Niven R. Narain, Michael A. Kiebish, Christine Ann Denny

**Affiliations:** 1 Division of Systems Neuroscience, Research Foundation for Mental Hygiene, Inc. (RFMH)/New York State Psychiatric Institute (NYSPI), New York, NY USA; 2 Department of Psychiatry, Columbia University Irving Medical Center (CUIMC), NYSPI Kolb Research Annex, Room 777, 1051 Riverside Drive, Unit 87, New York, NY USA; 3 BERG, Framingham, MA USA

Correction to: *Scientific Reports* 10.1038/s41598-020-68739-z, published online 22 July 2020

The Supplementary Information published with this Article contains errors, where Supplementary Tables S1–S8 were omitted. The corrected Supplementary Information file is linked to this correction notice.

## Supplementary information

Supplementary Information

