# Supplementary material for: Divergence in the metabolome between natural aging and Alzheimer’s disease
Source: Sci Rep. 2020 Jul 22;10:12171. doi: 10.1038/s41598-020-68739-z (PMC7376199; doi:10.1038/s41598-020-68739-z)
Supplement: Supplementary file 1 — Supplementary Information 1. [file 41598_2020_68739_MOESM1_ESM.docx]

**SUPPLEMENTAL INFORMATION**

**TITLE: Divergence in the metabolome between natural aging and Alzheimer’s disease**

**AUTHORS**: Holly C. Hunsberger^1,2^, Bennett P. Greenwood^3^, Vladimir Tolstikov^3^, Niven R. Narain^3^, Michael A. Kiebish^3^, and Christine A. Denny^1,2,*^

**SUPPLEMENTAL FIGURES AND FIGURE LEGENDS**

**Fig. S1** Correlation plots from Figure 2.

**a-d** In the PFC, 1-methylhistidine, l-cystathionine, l-tyrosine, and 3-methylhistidine are negatively correlated with freezing behavior in Ctrl mice. **e-g** 3-methylhistidine, tyrosine, and citrulline levels are negatively correlated with freezing behavior in Ctrl mice in the HPC. **h** The negative correlation between citrulline levels in the spleen and freezing behavior in Ctrl mice is not significant. **i-k** Carnosine, anserine, and isoleucine levels in the PFC are negatively correlated with freezing behavior in AD mice. **l-m** Anserine and carnosine levels are negatively correlated with freezing behavior in AD mice in the HPC. (n = 3-6 male mice per group). Ctrl, control; AD, Alzheimer’s disease; PFC, prefrontal cortex; HPC, hippocampus.

**Fig. S2** Histidine metabolism plots in Alzheimer’s disease mice.

**a** PFC levels of carnosine increased in 24-month-old AD mice when compared to 6- and 12-month-old AD mice. **b** PFC levels of formiminoglutamic acid were significantly decreased in 24-month-old AD mice when compared to 6- month-old AD mice. (C) HPC levels of carnosine increased in 24-month-old AD mice when compared to 6- and 12-month-old AD mice. **d-e** HPC levels of imazoleactic acid and 3-methylhistidine were significantly decreased in 24-month-old AD mice when compared to 6- month-old AD mice. **f** At 12 months of age, AD mice exhibited greater HPC levels of aspartic acid when compared to 6 and 24 months of age. **g-i** There were no significant differences in urocanic acid, histidine, or aspartic acid across ages in the spleens of AD mice. (n = 3-6 male mice per group). Error bars represent ± SEM. * p < 0.05; ** p < 0.01; *** p < 0.001. Ctrl, control; AD, Alzheimer’s disease; PFC, prefrontal cortex; HPC, hippocampus.

**Table S1.** CFC statistical analysis

**Table S2.** Tissue weights

**Table S3.** Histidine statistical analysis across tissue samples

**Table S4.** Prefrontal cortex statistical analysis

**Table S5.** Hippocampus statistical analysis

**Table S6.** Prefrontal cortex pathway analysis. The pathways in blue indicate the most significant pathways that are changed and are represented in the supplemental figure graphs.

**Table S7.** Hippocampus pathway analysis. The pathways in blue indicate the most significant pathways that are changed and are represented in the supplemental figure graphs.

**Table S8.** Spleen statistical analysis
