# Supplementary material for: Author Correction: Divergence in the metabolome between natural aging and Alzheimer’s disease
Source: Sci Rep. 2020 Nov 10;10:19863. doi: 10.1038/s41598-020-75425-7 (PMC7655935; doi:10.1038/s41598-020-75425-7)
Supplement: Supplementary file 1 — Supplementary Information [file 41598_2020_75425_MOESM1_ESM.pdf]

SUPPLEMENTAL INFORMATION

TITLE: Divergence in the metabolome between natural aging and Alzheimer's disease

AUTHORS: Holly Hunsberger[1,2], Bennett P. Greenwood[3], Vladimir Tolstikov[3], Niven R. Narain[3], Michael A. Kiebish[3], and Christine A. Denny[1,2,*]

SUPPLEMENTAL FIGURES AND FIGURE LEGENDS

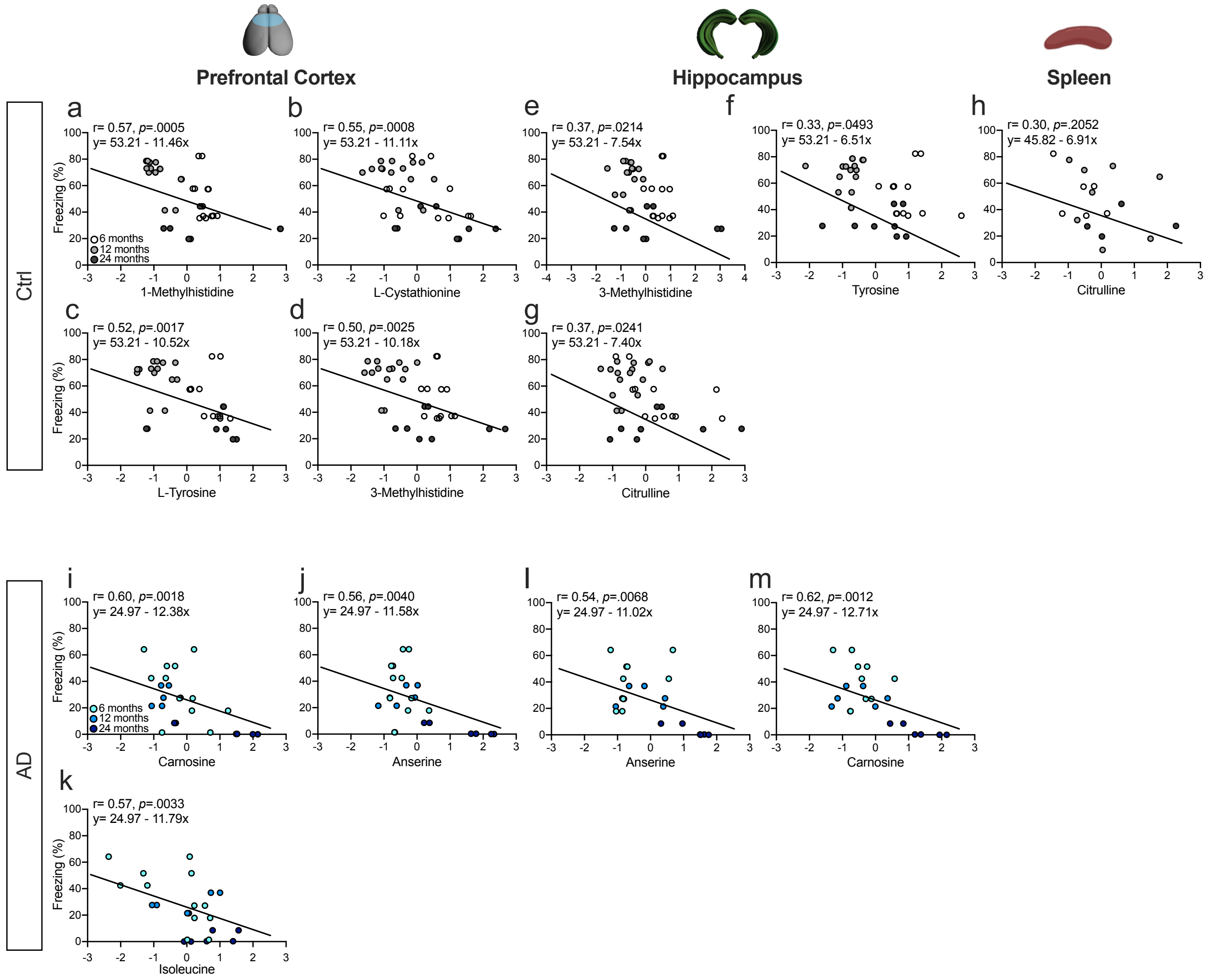

Fig. S1 Correlation plots from Figure 2.

a-d In the PFC, 1-methylhistidine, l-cystathionine, l-tyrosine, and 3-methylhistidine are negatively correlated with freezing behavior in Ctrl mice. **e-g** 3-methylhistidine, tyrosine, and citrulline levels are negatively correlated with freezing behavior in Ctrl mice in the HPC. **h** The negative correlation between citrulline levels in the spleen and freezing behavior in Ctrl mice is not significant. **i-k** Carnosine, anserine, and isoleucine levels in the PFC are negatively correlated with freezing behavior in AD mice. **l-m** Anserine and carnosine levels are negatively correlated with freezing behavior in AD mice in the HPC.

(n = 3-6 male mice per group). Ctrl, control; AD, Alzheimer’s disease; PFC, prefrontal cortex; HPC, hippocampus.

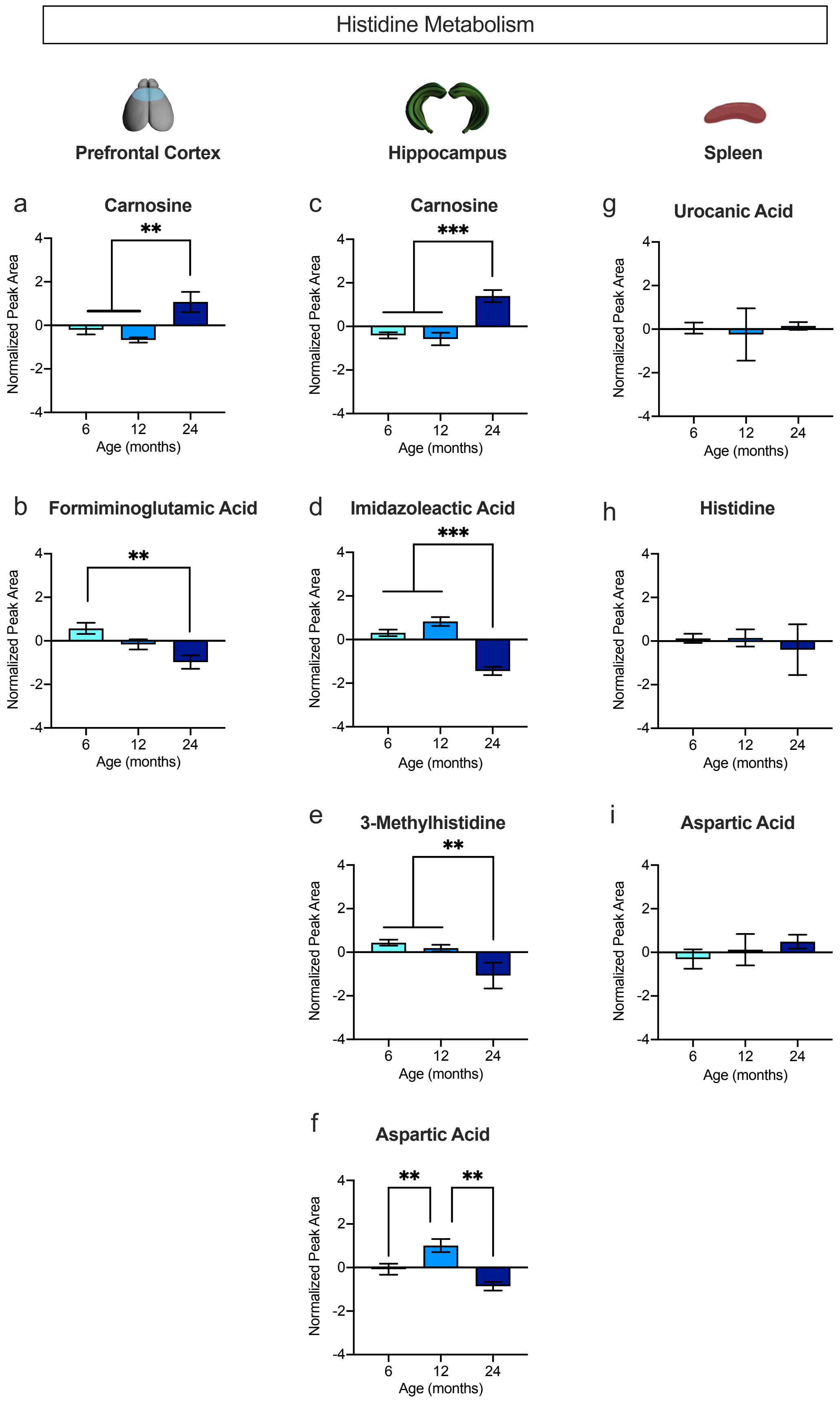

Histidine Metabolism
Prefrontal Cortex
Hippocampus
Spleen
a
Carnosine
**
Normalized Peak Area
Age (months)
c
Carnosine
***
g
Urocanic Acid
b
Formiminoglutamic Acid
**
d
Imidazoleactic Acid
***
h
Histidine
e
3-Methylhistidine
**
i
Aspartic Acid
f
Aspartic Acid
** **

Fig. S2 Histidine metabolism plots in Alzheimer's disease mice.

a PFC levels of carnosine increased in 24-month-old AD mice when compared to 6- and 12-month-old AD mice. **b** PFC levels of formiminoglutamic acid were significantly decreased in 24-month-old AD mice when compared to 6- month-old AD mice. (C) HPC levels of carnosine increased in 24-month-old AD mice when compared to 6- and 12-month-old AD mice. **d-e** HPC levels of imazoleactic acid and 3-methylhistidine were significantly decreased in 24-month-old AD mice when compared to 6- month-old AD mice. **f** At 12 months of age, AD mice exhibited greater HPC levels of aspartic acid when compared to 6 and 24 months of age. **g-i** There were no significant differences in urocanic acid, histidine, or aspartic acid across ages in the spleens of AD mice. (n = 3-6 male mice per group). Error bars represent ± SEM. * $p < 0.05$; ** $p < 0.01$; *** $p < 0.001$. Ctrl, control; AD, Alzheimer's disease; PFC, prefrontal cortex; HPC, hippocampus.

Table S1. CFC statistical analysis

Table S2. Tissue weights

Table S3. Histidine statistical analysis across tissue samples

Table S4. Prefrontal cortex statistical analysis

Table S5. Hippocampus statistical analysis

Table S6. Prefrontal cortex pathway analysis. The pathways in blue indicate the most significant pathways that are changed and are represented in the supplemental figure graphs.

Table S7. Hippocampus pathway analysis. The pathways in blue indicate the most significant pathways that are changed and are represented in the supplemental figure graphs.

Table S8. Spleen statistical analysis

Table S1. CFC statistical analysis

Behavioral Paradigm	Day	Statistical test	Comparison	F	° of freedom	p	*	Fig.
CFC	Day 1	RMANOVA	Age	0.8853	2,25	0.4300	-	data not included
			Genotype	0.8234	1,25	0.3700	-	
			Age x Genotype	0.9216	2,25	0.4100	-	
			Time	4.7723	4,22	0.0063	**	
			Time x Age	0.7476	8,44	0.6500	-	
			Time x Genotype	1.9974	4,22	0.1300	-	
			Time x Age x Genotype	1.4168	8,44	0.2200	-	
	Day 2	ANOVA	Age	9.889	5,37	0.0005	**	1b
			Genotype	16.6954	5,37	0.0003	**	
			Age x Genotype	0.3094	5,37	0.7360	-	
		post-hoc *t*-tests	Ctrl 6 x Ctrl 12	-	-	0.9091	-	
			Ctrl 6 x Ctrl 24	-	-	0.0037	**	
			Ctrl 12 x Ctrl 24	-	-	0.0053	**	
			AD 6 x AD 12	-	-	0.3095	-	
			AD 6 x AD 24	-	-	0.0036	**	
			AD 12 x AD 24	-	-	0.0939	-	
			Ctrl 6 x AD 6	-	-	0.0122	*	
			Ctrl 12 x AD 12	-	-	0.0089	**	
			Ctrl 24 x AD 24	-	-	0.0632	-	

Table S2. Tissue weights

Group	Genotype	Prefrontal Cortex (mg)		Hippocampus (mg)		Spleen (mg)
		Left	Right	Left	Right	
6-month-old mice	Ctrl	18.90	17.70	16.70	32.80	60.20
	Ctrl	15.00	20.80	15.30	18.40	56.40
	Ctrl	21.30	22.80	21.10	22.50	90.10
	Ctrl	24.00	23.00	15.10	20.20	67.80
	Ctrl	20.10	24.40	17.50	21.10	82.50
	Ctrl	18.40	14.60	27.40	20.80	46.50
	AD	8.20	7.30	27.70	20.00	115.60
	AD	7.20	10.20	19.40	22.20	80.60
	AD	5.00	5.10	15.50	15.30	74.20
	AD	7.40	6.40	12.40	13.20	150.30
	AD	5.40	7.70	15.20	16.80	121.60
	AD	4.50	6.60	18.70	20.20	122.20
1-year-old mice	Ctrl	3.50	16.00	15.40	16.00	67.00
	Ctrl	14.10	16.60	12.60	13.00	121.30
	Ctrl	17.50	13.10	15.50	12.70	94.40
	Ctrl	14.80	14.50	16.60	13.30	81.60
	Ctrl	13.00	11.40	18.00	13.70	100.70
	Ctrl	16.70	16.90	10.60	12.40	80.60
	AD	12.80	11.40	16.10	36.70	612.90
	AD	16.50	14.50	17.30	29.80	146.20
	AD	16.20	14.60	17.50	19.40	92.70
2-year-old mice	Ctrl	19.30	18.20	20.20	16.60	199.80
	Ctrl	21.40	25.90	19.10	12.50	112.50
	Ctrl	13.30	9.00	9.80	9.30	185.10
	Ctrl	23.70	27.20	15.00	19.50	207.50
	AD	10.50	11.00	18.10	14.80	35.80
	AD	26.20	22.90	28.40	18.50	43.90
	AD	8.30	9.10	22.90	14.20	97.60

Table S3. Histidine statistical analysis across tissue samples

Tissue	Metabolite	Statistical test	Comparison	F	° of freedom	p	*	Fig.
Prefrontal Cortex	Carnosine	ANOVA	Age	8.2337	2,23	0.0023	**	S2a
		post-hoc *t*-tests	AD 6 x AD 12	-	-	0.2452	-	
			AD 6 x AD 24	-	-	0.0038	**	
			AD 12 x AD 24	-	-	0.0009	**	
	Formiminoglutamic Acid	ANOVA	Age	7.8399	2,23	0.0029	**	S2b
		post-hoc *t*-tests	AD 6 x AD 12	-	-	0.0782	-	
			AD 6 x AD 24	-	-	0.0008	**	
			AD 12 x AD 24	-	-	0.0880	-	
Hippocampus	Carnosine	ANOVA	Age	22.3648	2,23	0.0001	***	S2c
		post-hoc *t*-tests	AD 6 x AD 12	-	-	0.5738	-	
			AD 6 x AD 24	-	-	0.0001	***	
			AD 12 x AD 24	-	-	0.0001	***	
	Imidazoleactic Acid	ANOVA	Age	35.0997	2,23	0.0001	***	S2d
		post-hoc *t*-tests	AD 6 x AD 12	-	-	0.0509	-	
			AD 6 x AD 24	-	-	0.0001	***	
			AD 12 x AD 24	-	-	0.0001	***	
	L-Aspartic Acid	ANOVA	Age	9.0312	2,23	0.0015	**	S2e
		post-hoc *t*-tests	AD 6 x AD 12	-	-	0.0099	**	
			AD 6 x AD 24	-	-	0.0541	-	
			AD 12 x AD 24	-	-	0.0004	**	
	3-Methylhistidine	ANOVA	Age	7.2859	2,23	0.0040	**	S2f
		post-hoc *t*-tests	AD 6 x AD 12	-	-	0.5431	-	
			AD 6 x AD 24	-	-	0.0012	**	
			AD 12 x AD 24	-	-	0.0129	*	
Spleen	Urocanic Acid	ANOVA	Age	0.1082	2,11	0.8986	-	S2g
	Histidine	ANOVA	Age	0.2690	2,11	0.7701	-	S2h
	Aspartic Acid	ANOVA	Age	0.6238	2,11	0.5576	-	S2i

Table S4. Prefrontal cortex statistical analysis

Tissue	Metabolite	Statistical test	Comparison	F	° of freedom	p	*	Fig.
	S-Adenosylmethionine	ANOVA	Age	15.5741	2,57	0.0001	***	4b
			Genotype	34.7710	1,57	0.0001	***	
			Age x Genotype	0.9143	2,57	0.4072	-	
		Tukey HSD	Ctrl 6 x Ctrl 12	-	-	0.0001	***	
			Ctrl 6 x Ctrl 24	-	-	0.0263	*	
			Ctrl 12 x Ctrl 24	-	-	0.6756	-	
			AD 6 x AD 12	-	-	0.1169	-	
			AD 6 x AD 24	-	-	0.4954	-	
			AD 12 x AD 24	-	-	0.9790	-	
			Ctrl 6 x AD 6	-	-	0.0001	***	
			Ctrl 12 x AD 12	-	-	0.1077	-	
			Ctrl 24 x AD 24	-	-	0.0684	-	
	4-Guanidinobutanoic Acid	ANOVA	Age	0.7983	2,57	0.4555	-	4c
			Genotype	1.1893	1,57	0.2805	-	
			Age x Genotype	12.2324	2,57	0.0001	***	
		Tukey HSD	Ctrl 6 x Ctrl 12	-	-	0.0004	**	
			Ctrl 6 x Ctrl 24	-	-	0.1187	-	
			Ctrl 12 x Ctrl 24	-	-	0.7306	-	
			AD 6 x AD 12	-	-	0.2710	-	
			AD 6 x AD 24	-	-	0.1454	-	
			AD 12 x AD 24	-	-	0.9997	-	
			Ctrl 6 x AD 6	-	-	0.0090	**	
			Ctrl 12 x AD 12	-	-	0.0576	-	
			Ctrl 24 x AD 24	-	-	0.4878	-	
	Argininosuccinic Acid	ANOVA	Age	10.6047	2,57	0.0001	***	4d
			Genotype	0.1215	1,57	0.7288	-	
			Age x Genotype	9.7307	2,57	0.0003	**	
		Tukey HSD	Ctrl 6 x Ctrl 12	-	-	0.0001	***	
			Ctrl 6 x Ctrl 24	-	-	0.3392	-	
			Ctrl 12 x Ctrl 24	-	-	0.1438	-	
			AD 6 x AD 12	-	-	0.9992	-	
			AD 6 x AD 24	-	-	0.0048	**	
			AD 12 x AD 24	-	-	0.0087	**	
			Ctrl 6 x AD 6	-	-	0.0022	**	

			Ctrl 12 x AD 12	-	-	0.9985	-	
			Ctrl 24 x AD 24	-	-	0.2702	-	
	N-Acetyl-L-aspartic Acid	ANOVA	Age	2.2607	2,57	0.1144	-	4e
			Genotype	3.9887	1,57	0.0511	-	
			Age x Genotype	7.1847	2,57	0.0018	**	
		Tukey HSD	Ctrl 6 x Ctrl 12	-	-	0.0004	**	
			Ctrl 6 x Ctrl 24	-	-	0.1471	-	
			Ctrl 12 x Ctrl 24	-	-	0.6450	-	
			AD 6 x AD 12	-	-	0.8468	-	
			AD 6 x AD 24	-	-	1.0000	-	
			AD 12 x AD 24	-	-	0.9460	-	
			Ctrl 6 x AD 6	-	-	0.0010	**	
			Ctrl 12 x AD 12	-	-	0.7732	-	
			Ctrl 24 x AD 24	-	-	0.9081	-	
PFC	Phenylpyruvic-acid	ANOVA	Age	21.4272	2,57	0.0001	***	4f
			Genotype	32.4515	1,57	0.0001	***	
			Age x Genotype	5.1704	2,57	0.0090	**	
		Tukey HSD	Ctrl 6 x Ctrl 12	-	-	0.0001	***	
			Ctrl 6 x Ctrl 24	-	-	0.8395	-	
			Ctrl 12 x Ctrl 24	-	-	0.0001	***	
			AD 6 x AD 12	-	-	0.3117	-	
			AD 6 x AD 24	-	-	0.9971	-	
			AD 12 x AD 24	-	-	0.7270	-	
			Ctrl 6 x AD 6	-	-	0.0001	***	
			Ctrl 12 x AD 12	-	-	0.9738	-	
			Ctrl 24 x AD 24	-	-	0.0004	**	
	L-Tyrosine	ANOVA	Age	14.6609	2,57	0.0001	***	4g
			Genotype	17.8027	1,57	0.0001	***	
			Age x Genotype	3.3295	2,57	0.0436	*	
		Tukey HSD	Ctrl 6 x Ctrl 12			0.0001	***	
			Ctrl 6 x Ctrl 24	-	-	0.9999	-	
			Ctrl 12 x Ctrl 24	-	-	0.0001	***	
			AD 6 x AD 12	-	-	0.5247	-	
			AD 6 x AD 24	-	-	1.0000	-	
			AD 12 x AD 24	-	-	0.7608	-	
			Ctrl 6 x AD 6	-	-	0.0017	**	
			Ctrl 12 x AD 12	-	-	0.9993	-	
			Ctrl 24 x AD 24	-	-	0.0368	*	

1-Methylhistamine	ANOVA	Age	6.1051	2,57	0.0042	**	4h
		Genotype	4.4428	1,57	0.0399	*	
		Age x Genotype	7.4014	2,57	0.0015	**	
	Tukey HSD	Ctrl 6 x Ctrl 12	-	-	0.0001	***	
		Ctrl 6 x Ctrl 24	-	-	0.9834	-	
		Ctrl 12 x Ctrl 24	-	-	0.0001	***	
		AD 6 x AD 12	-	-	0.9979	-	
		AD 6 x AD 24	-	-	0.7473	-	
		AD 12 x AD 24	-	-	0.9651	-	
		Ctrl 6 x AD 6	-	-	0.3375	-	
		Ctrl 12 x AD 12	-	-	0.0410	-	
		Ctrl 24 x AD 24	-	-	0.0166	*	
1-Methylhistidine	ANOVA	Age	6.9155	2,57	0.0020	**	4i
		Genotype	2.0712	1,57	0.1561	-	
		Age x Genotype	9.1003	2,57	0.0004	**	
	Tukey HSD	Ctrl 6 x Ctrl 12	-	-	0.0001	***	
		Ctrl 6 x Ctrl 24	-	-	0.9959	-	
		Ctrl 12 x Ctrl 24	-	-	0.0001	***	
		AD 6 x AD 12	-	-	0.9985	-	
		AD 6 x AD 24	-	-	0.5441	-	
		AD 12 x AD 24	-	-	0.8743	-	
		Ctrl 6 x AD 6	-	-	0.5498	-	
		Ctrl 12 x AD 12	-	-	0.1276	-	
		Ctrl 24 x AD 24	-	-	0.0229	*	
Formiminoglutamic Acid	ANOVA	Age	1.8970	2,57	0.1603	-	4j
		Genotype	49.2604	1,57	0.0001	***	
		Age x Genotype	17.1620	2,57	0.0001	***	
	Tukey HSD	Ctrl 6 x Ctrl 12	-	-	0.9988	-	
		Ctrl 6 x Ctrl 24	-	-	0.0089	**	
		Ctrl 12 x Ctrl 24	-	-	0.0023	**	
		AD 6 x AD 12	-	-	0.2751	-	
		AD 6 x AD 24	-	-	0.0004	**	
		AD 12 x AD 24	-	-	0.3109	-	
		Ctrl 6 x AD 6	-	-	0.9620	-	
		Ctrl 12 x AD 12	-	-	0.1192	-	
		Ctrl 24 x AD 24	-	-	0.0001	***	
	ANOVA	Age	4.9363	2,57	0.0109	*	
		Genotype	8.9649	1,57	0.0042	**	

	Phosphocreatine		Age x Genotype	13.4663	2,57	0.0001	***	4k
		Tukey HSD	Ctrl 6 x Ctrl 12	-	-	0.0001	***	
			Ctrl 6 x Ctrl 24	-	-	0.7170	-	
			Ctrl 12 x Ctrl 24	-	-	0.0001	***	
			AD 6 x AD 12	-	-	0.3397	-	
			AD 6 x AD 24	-	-	0.8032	-	
			AD 12 x AD 24	-	-	0.9851	-	
			Ctrl 6 x AD 6	-	-	0.0005	**	
			Ctrl 12 x AD 12	-	-	0.1567	-	
			Ctrl 24 x AD 24	-	-	0.0141	*	

Table S5. Hippocampus statistical analysis

Tissue	Metabolite	Statistical test	Comparison	F	° of freedom	p	*	Fig.
	L-Methionine	ANOVA	Age	16.9227	2,59	0.0001	***	5b
			Genotype	17.8804	1,59	0.0001	***	
			Age x Genotype	3.4294	2,59	0.0396	*	
		Tukey HSD	Ctrl 6 x Ctrl 12	-	-	0.0030	**	
			Ctrl 6 x Ctrl 24	-	-	0.0001	***	
			Ctrl 12 x Ctrl 24	-	-	0.1863	-	
			AD 6 x AD 12	-	-	0.0465	*	
			AD 6 x AD 24	-	-	0.4979	-	
			AD 12 x AD 24	-	-	0.8958	-	
			Ctrl 6 x AD 6	-	-	0.0009	**	
			Ctrl 12 x AD 12	-	-	0.0051	**	
			Ctrl 24 x AD 24	-	-	1.0000	-	
	L-Tyrosine	ANOVA	Age	17.7812	2,59	0.0001	***	5c
			Genotype	20.6768	1,59	0.0001	***	
			Age x Genotype	2.5492	2,59	0.0875	-	
		Tukey HSD	Ctrl 6 x Ctrl 12	-	-	0.0001	***	
			Ctrl 6 x Ctrl 24	-	-	0.0346	*	
			Ctrl 12 x Ctrl 24	-	-	0.3377	-	
			AD 6 x AD 12	-	-	0.2550	-	
			AD 6 x AD 24	-	-	0.4198	-	
			AD 12 x AD 24	-	-	0.9998	-	
			Ctrl 6 x AD 6	-	-	0.0002	**	
			Ctrl 12 x AD 12	-	-	0.8495	-	
			Ctrl 24 x AD 24	-	-	0.1942	-	
	Glyceric Acid	ANOVA	Age	18.8428	2,59	0.0001	***	5d
			Genotype	50.4089	1,59	0.0001	***	
			Age x Genotype	3.8042	2,59	0.0285	*	
		Tukey HSD	Ctrl 6 x Ctrl 12	-	-	0.9961	-	
			Ctrl 6 x Ctrl 24	-	-	0.0398	*	
			Ctrl 12 x Ctrl 24	-	-	0.0666	-	
			AD 6 x AD 12	-	-	0.0073	**	
			AD 6 x AD 24	-	-	0.0001	***	
			AD 12 x AD 24	-	-	0.5957	-	
			Ctrl 6 x AD 6	-	-	0.1212	-	
			Ctrl 12 x AD 12	-	-	0.0001	***	

			Ctrl 24 x AD 24	-	-	0.0029	**	
HPC	L-Isoleucine	ANOVA	Age	4.4912	2,59	0.0157	*	5e
			Genotype	0.0313	1,59	0.8601	-	
			Age x Genotype	3.5415	2,59	0.0359	*	
		Tukey HSD	Ctrl 6 x Ctrl 12	-	-	0.0639	-	
			Ctrl 6 x Ctrl 24	-	-	0.0210	*	
			Ctrl 12 x Ctrl 24	-	-	0.8213	-	
			AD 6 x AD 12	-	-	0.8964	-	
			AD 6 x AD 24	-	-	0.9360	-	
			AD 12 x AD 24	-	-	0.5232	-	
			Ctrl 6 x AD 6	-	-	0.1807	-	
			Ctrl 12 x AD 12	-	-	0.7766	-	
			Ctrl 24 x AD 24	-	-	0.9992	-	
	Hydroxypyruvic Acid	ANOVA	Age	44.4945	2,59	0.0001	***	5f
			Genotype	22.6033	1,59	0.0001	***	
			Age x Genotype	15.7293	2,59	0.0001	***	
		Tukey HSD	Ctrl 6 x Ctrl 12	-	-	0.0004	**	
			Ctrl 6 x Ctrl 24	-	-	0.0001	***	
			Ctrl 12 x Ctrl 24	-	-	0.0010	**	
			AD 6 x AD 12	-	-	0.5607	-	
			AD 6 x AD 24	-	-	0.0096	**	
			AD 12 x AD 24	-	-	0.5746	-	
			Ctrl 6 x AD 6	-	-	0.0001	***	
			Ctrl 12 x AD 12	-	-	0.0089	**	
			Ctrl 24 x AD 24	-	-	0.8896	-	
	Pyroglutamic Acid	ANOVA	Age	31.1982	2,59	0.0001	***	5g
			Genotype	33.0849	1,59	0.0001	***	
			Age x Genotype	3.9084	2,59	0.0260	*	
		Tukey HSD	Ctrl 6 x Ctrl 12	-	-	0.0001	***	
			Ctrl 6 x Ctrl 24	-	-	0.0001	***	
			Ctrl 12 x Ctrl 24	-	-	0.0001	***	
			AD 6 x AD 12	-	-	0.1018	-	
			AD 6 x AD 24	-	-	0.0008	**	
			AD 12 x AD 24	-	-	0.1063	-	
			Ctrl 6 x AD 6	-	-	0.0001	***	
			Ctrl 12 x AD 12	-	-	0.0007	**	
			Ctrl 24 x AD 24	-	-	0.2876	-	
			Age	22.0532	2,59	0.0001	***	

	Glycolic Acid	ANOVA	Genotype	6.8814	1,59	0.0100	*	5h
			Age x Genotype	6.8894	2,59	0.0022	**	
		Tukey HSD	Ctrl 6 x Ctrl 12	-	-	0.0014	**	
			Ctrl 6 x Ctrl 24	-	-	0.0001	***	
			Ctrl 12 x Ctrl 24	-	-	0.0023	**	
			AD 6 x AD 12	-	-	0.1391	-	
			AD 6 x AD 24	-	-	0.4424	-	
			AD 12 x AD 24	-	-	0.9925	-	
			Ctrl 6 x AD 6	-	-	0.0016	**	
			Ctrl 12 x AD 12	-	-	0.0524	-	
			Ctrl 24 x AD 24			0.6855	-	
	Arginine	ANOVA	Age	11.3143	2,59	0.0001	***	5i
			Genotype	18.1422	1,59	0.0001	***	
			Age x Genotype	0.9923	2,59	0.3774	-	
		Tukey HSD	Ctrl 6 x Ctrl 12	-	-	0.7768	-	
			Ctrl 6 x Ctrl 24	-	-	0.0041	**	
			Ctrl 12 x Ctrl 24	-	-	0.0403	*	
			AD 6 x AD 12	-	-	0.3407	-	
			AD 6 x AD 24	-	-	0.0704	-	
			AD 12 x AD 24	-	-	0.9833	-	
			Ctrl 6 x AD 6	-	-	0.0509	-	
			Ctrl 12 x AD 12	-	-	0.0071	**	
			Ctrl 24 x AD 24	-	-	0.8385	-	
	Glutathione	ANOVA	Age	6.7371	2,59	0.0024	**	5j
			Genotype	1.1824	1,59	0.2817	-	
			Age x Genotype	6.1361	2,59	0.0040	**	
		Tukey HSD	Ctrl 6 x Ctrl 12	-	-	0.5559	-	
			Ctrl 6 x Ctrl 24	-	-	0.0015	**	
			Ctrl 12 x Ctrl 24	-	-	0.0340	*	
			AD 6 x AD 12	-	-	0.1645	-	
			AD 6 x AD 24	-	-	0.9998	-	
			AD 12 x AD 24	-	-	0.4292	-	
			Ctrl 6 x AD 6	-	-	0.9992	-	
			Ctrl 12 x AD 12	-	-	0.6245	-	
			Ctrl 24 x AD 24	-	-	0.0318	*	

Table S6. Prefrontal cortex pathway analysis

Pathway name	Metabolites	Ctrl vs AD	Age (mo)	p	-LOG(p)	Impact
Lysine Biosynthesis	Saccharopine		6	0.0021	6.19	0
	Aminoadipic Acid		6	0.0021	6.19	0
Phenylalanine, tyrosine, and tryptophan	Phenylpyruvic Acid	Increased	6	0.0021	6.19	0.5
	L-Tyrosine	Increased	6	0.0021	6.19	0.5
Histidine metabolism	1-Methylhistamine	Increased	6	0.0024	6.03	0.11
	1-Methylhistidine	Increased	6	0.0024	6.03	0.11
Arginine and proline	Argininosuccinic Acid	Increased	6	0.0084	4.78	0.12
	S-Adenosylmethionine	Increased	6	0.0084	4.78	0.12
	Phosphocreatine	Decreased	6	0.0084	4.78	0.12
Alanine, asparate, and glutamate	N-Acetyl-L-Aspartic Acid	Increased	6	0.0096	4.65	0.28
	Argininosuccinic Acid	Increased	6	0.0096	4.65	0.28
Phenylalanine	Phenylpyruvic Acid		6	0.0173	4.06	0.24
	L-Tyrosine		6	0.0173	4.06	0.24
Ubiquinone and other terpenoid-quione Biosynthesis	L-Tyrosine		6	0.0561	2.88	0
Lysine Degradation	L-Tyrosine		6	0.0692	2.67	0.01
D-Glutamine and D-glutamate	L-Glutamic Acid		6	0.0918	2.39	1
Taurine and hypotaurine	Hypotaurine		6	0.1430	1.95	0.29
Nitrogen	L-Glutamic Acid		6	0.1594	1.84	0
Pyrimidine	Uridine 5' - diphosphate		6	0.1824	1.7	0.11
	Cytidine-5'-diphosphate		6	0.1824	1.7	0.11
Pentose and gluccuronate interconversions	L-Arabinose		6	0.2662	1.32	0
Glyoxylate and dicarboxylate	Glyceric Acid		6	0.2942	1.22	0.06
Glycerolipid	Glyceric Acid		6	0.2942	1.22	0.1
Fructose and mannose	Sorbitol		6	0.3343	1.1	0.03
Butanoate	L-Glutamic Acid		6	0.3472	1.06	0
Aminoacyl-tRNA Biosynthesis	L-Tyrosine		6	0.3821	0.96	0
	L-Glutamic Acid		6	0.3821	0.96	0
Galactose	Sorbitol		6	0.3963	0.93	0
Glutathione	L-Glutamic Acid		6	0.3963	0.93	0.06
Porphyrin and chlorophyll metabolism	L-Glutamic Acid		6	0.4080	0.90	0.00
Cysteine and methionine metabolism	S-Adenosylmethionine		6	0.4080	0.90	0.07
Glycine, serine and threonine metabolism	Glyceric Acid		6	0.4528	0.79	0.00

Amino sugar and nucleotide sugar metabolism	Uridine diphosphate N-acetylglucosamine		6	0.5138	0.67	0.01
Biosynthesis of unsaturated fatty acids	7,10,13,16-Docosatetraenoic acid		6	0.5596	0.58	0.00
Tyrosine metabolism	L-Tyrosine		6	0.5767	0.55	0.14
Purine metabolism	Guanosine monophosphate		6	0.7383	0.30	0.04
Histidine metabolism	1-Methylhistamine	Decreased	12	0.0033	5.82	0.17
	Formiminoglutamic Acid	Increased	12	0.0033	5.82	0.17
	1-Methylhistidine	Decreased	12	0.0033	5.82	0.17
Glyoxylate and dicarboxylate metabolism	Hydroxypyruvic Acid		12	0.0056	5.27	0.16
	Glycolic Acid		12	0.0056	5.27	0.16
	Glyceric Acid		12	0.0056	5.27	0.16
Glycine, serine and threonine metabolism	Glyceric Acid		12	0.0258	3.75	0.09
	Phosphoserine		12	0.0258	3.75	0.09
	Hydroxypyruvic Acid		12	0.0258	3.75	0.09
Pentose phosphate pathway	Glucose 6-phosphate		12	0.0593	2.89	0.07
	D-Ribose		12	0.0593	2.89	0.07
Glycolysis or Gluconeogenesis	L-Lactic Acid		12	0.1029	2.33	0.04
	Glucose 6-phosphate		12	0.1029	2.33	0.04
Pyrimidine metabolism	Dihydrothymine		12	0.2145	1.59	0.07
	Uracil		12	0.2145	1.59	0.07
Nicotinate and nicotinamide metabolism	Nicotinamide adenine dinucleotide		12	0.2438	1.44	0.21
Pantothenate and CoA biosynthesis	Uracil		12	0.2757	1.32	0.00
beta-Alanine metabolism	Uracil		12	0.3064	1.21	0.00
Glycerolipid metabolism	Glyceric Acid		12	0.3213	1.16	0.10
Starch and sucrose metabolism	Glucose 6-phosphate		12	0.3358	1.12	0.17
Pyruvate metabolism	L-Lactic Acid		12	0.3911	0.97	0.00
Purine metabolism	Adenosine Triphosphate		12	0.4269	0.89	0.02
	Hypoxanthine		12	0.4269	0.89	0.02
Glutathione metabolism	Pyroglutamic Acid		12	0.4296	0.87	0.01
Galactose metabolism	Glucose 6-phosphate		12	0.4296	0.87	0.02
Glycerolipid metabolism	Glycerophosphocholine		12	0.4773	0.74	0.02
Amino sugar and nucleotide sugar metabolism	Glucose 6-phosphate		12	0.5516	0.62	0.09
Tryptophan metabolism	3-Hydroxyanthranilic Acid		12	0.5803	0.57	0.05

Arginine and proline metabolism	4-Guanidinobutanoic Acid	Decreased	12	0.6157	0.51	0
Nicotinate and nicotinamide metabolism	Nicotinamide adenine dinucleotide		24	0.0451	3.10	0.21
Histidine metabolism	Formiminoglutamic Acid	Increased	24	0.0519	2.96	0.06
Biosynthesis of unsaturated fatty acids	7,10,13,16-Docosatetraenoic acid		24	0.1399	1.97	0.00

Table S7. Hippocampus pathway analysis

Pathway name	Metabolites	Ctrl vs AD	Age (mo)	p	-LOG(p)	Impact
Aminoacyl-tRNAbiosynthesis	L-Methionine	Increased	6	0.0222	3.81	0.00
	L-Isoleucine	Increased	6	0.0222	3.81	0.00
	L-Tyrosine	Increased	6	0.0222	3.81	0.00
Glutathione metabolism	Glutathione	Increased	6	0.0223	3.80	0.38
	Pyroglutamic acid	Decreased	6	0.0223	3.80	0.38
Ubiquinone and other terpenoidquinone biosynthesis	L-tyrosine		6	0.0273	3.60	0.00
Glycine, serine, threonine metabolism	Hydroxypyruvic		6	0.0311	3.47	0.00
	Pyruvaldehyde		6	0.0362	3.32	0.50
Phenylalanine, tyrosine, and tryptophan biosynthesis	L-Tyrosine		6	0.0362	3.32	0.50
Taurine and hypotaurine metabolism	Taurine		6	0.0713	2.64	0.43
Phenylalanine metabolism	L-Tyrosine		6	0.0967	2.34	0.00
Valine, leucine, and isoleucine biosynthesis	L-Isoleucine		6	0.0967	2.34	0.33
Glyoxylate and dicarboxylate metabolism	Hydroxypyruvic acid	Decreased	6	0.1537	1.87	0.06
Pyruvate metabolism	Pyruvaldehyde		6	0.1924	1.65	0.07
Cysteine and methionine metabolism	L-methionine		6	0.2221	1.50	0.09
Valine, leucine, and isoleucine degredation	L-Isoleucine		6	0.2988	1.21	0.00
Tyrosine metabolism	L-Tyrosine		6	0.3376	1.09	0.14
Primary bile acid biosynthesis	Taurine		6	0.3501	1.05	0.03
Purine metabolism	Inosine		6	0.4738	0.75	0.00
Glyoxylate and dicarboxylate metabolism	Glycolic acid	Decreased	12	0.0109	4.51	0.10
	Glyceric acid	Increased	12	0.0109	4.51	0.10
Arginine and proline metabolism	Citrulline		12	0.0592	2.83	0.11
	L-Arginine		12	0.0592	2.83	0.11
Aminoacyl-tRNAbiosynthesis	L-Arginine	Decreased	12	0.1290	2.05	0.00
	L-Methionine	Increased	12	0.1290	2.05	0.00
Histidine metabolism	1-Methylhistidine		12	0.1297	2.04	0.00
Glycerolipid metabolism	Glyceric acid		12	0.1537	1.87	0.10

Pentose phosphate pathway	6-Phosphogluconic acid		12	0.1616	1.82	0.05
Glutathione metabolism	Pyroglutamic acid	Decreased	12	0.2148	1.54	0.01
Cysteine and methionine metabolism	L-methionine		12	0.2221	1.50	0.09
Glycine, serine, threonine metabolism	Glyceric acid		12	0.2508	1.38	0.00
Pyrimidine metabolism	Cytidine monophosphate		12	0.3184	1.14	0.01
Purine metabolism	Cyclic AMP		12	0.4738	0.75	0.00

Table S8. Spleen statistical analysis

Tissue	Metabolite	Statistical test	Comparison	F	° of freedom	p	*	Fig.
	Spleen weight	ANOVA	Age	3.3315	2,29	0.0529	-	6b
			Genotype	0.7214	1,29	0.4041	-	
			Age x Genotype	4.2178	2,29	0.0269	*	
		post-hoc *t*-tests	Ctrl 6 x Ctrl 12	-	-	0.1763	-	
			Ctrl 6 x Ctrl 24	-	-	0.0140	*	
			Ctrl 12 x Ctrl 24	-	-	0.1138	-	
			AD 6 x AD 12	-	-	0.1085	-	
			AD 6 x AD 24	-	-	0.6081	-	
			AD 12 x AD 24	-	-	0.0762	-	
			Ctrl 6 x AD 6	-	-	0.0090	**	
			Ctrl 12 x AD 12	-	-	0.1360	-	
			Ctrl 24 x AD 24	-	-	0.0120	*	
	n-Acetylneuraminic Acid	ANOVA	Age	2.1027	2,29	0.1441	-	6d
			Genotype	5.9491	1,29	0.0225	*	
			Age x Genotype	8.2969	2,29	0.0018	**	
		Tukey HSD	Ctrl 6 x Ctrl 12	-	-	0.8017	-	
			Ctrl 6 x Ctrl 24	-	-	0.1430	-	
			Ctrl 12 x Ctrl 24	-	-	0.6071	-	
			AD 6 x AD 12	-	-	0.7681	-	
			AD 6 x AD 24	-	-	0.1459	-	
			AD 12 x AD 24	-	-	0.0272	*	
			Ctrl 6 x AD 6	-	-	0.0197	*	
			Ctrl 12 x AD 12	-	-	0.0271	*	
			Ctrl 24 x AD 24	-	-	0.4293	-	
	Phosphoribosylaminoimidazolesuccinocarboxamide (SAICAR)	ANOVA	Age	5.2010	2,29	0.0133	*	6e
			Genotype	25.8441	1,29	0.0001	***	
			Age x Genotype	4.0917	2,29	0.0296	*	
		Tukey HSD	Ctrl 6 x Ctrl 12	-	-	0.0128	*	
			Ctrl 6 x Ctrl 24	-	-	0.4776	-	
			Ctrl 12 x Ctrl 24	-	-	0.7550	-	
			AD 6 x AD 12	-	-	0.9777	-	
			AD 6 x AD 24	-	-	0.3086	-	
			AD 12 x AD 24	-	-	0.1751	-	
			Ctrl 6 x AD 6	-	-	0.0001	***	
			Ctrl 12 x AD 12	-	-	0.0946	-	

			Ctrl 24 x AD 24	-	-	0.9378	-
Orotate	ANOVA	Age	9.1510	2,29	0.0011	**	6f
		Genotype	19.8452	1,29	0.0002	**	
		Age x Genotype	1.1914	2,29	0.3211	-	
	Tukey HSD	Ctrl 6 x Ctrl 12	-	-	0.0189	*	
		Ctrl 6 x Ctrl 24	-	-	0.9724	-	
		Ctrl 12 x Ctrl 24	-	-	0.2265	-	
		AD 6 x AD 12	-	-	0.3912	-	
		AD 6 x AD 24	-	-	0.7264	-	
		AD 12 x AD 24	-	-	0.0730	-	
		Ctrl 6 x AD 6	-	-	0.0041	**	
		Ctrl 12 x AD 12	-	-	0.0938	-	
		Ctrl 24 x AD 24	-	-	0.8249	-	
Uridine	ANOVA	Age	3.4128	2,29	0.0496	*	6g
		Genotype	8.7963	1,29	0.0067	**	
		Age x Genotype	1.0228	2,29	0.3747	-	
	Tukey HSD	Ctrl 6 x Ctrl 12	-	-	0.9785	-	
		Ctrl 6 x Ctrl 24	-	-	0.9650	-	
		Ctrl 12 x Ctrl 24	-	-	0.6854	-	
		AD 6 x AD 12	-	-	0.9886	-	
		AD 6 x AD 24	-	-	0.1445	-	
		AD 12 x AD 24	-	-	0.5665	-	
		Ctrl 6 x AD 6	-	-	0.0359	*	
		Ctrl 12 x AD 12	-	-	0.6528	-	
		Ctrl 24 x AD 24	-	-	0.9732	-	
Propionylcarnitine	ANOVA	Age	1.8719	2,29	0.1756	-	6h
		Genotype	0.0921	1,29	0.7642	-	
		Age x Genotype	6.1287	2,29	0.0071	**	
	Tukey HSD	Ctrl 6 x Ctrl 12	-	-	0.0963	-	
		Ctrl 6 x Ctrl 24	-	-	0.8779	-	
		Ctrl 12 x Ctrl 24	-	-	0.7858	-	
		AD 6 x AD 12	-	-	0.6899	-	
		AD 6 x AD 24	-	-	0.0553	-	
		AD 12 x AD 24	-	-	0.7435	-	
		Ctrl 6 x AD 6	-	-	0.0303	*	
		Ctrl 12 x AD 12	-	-	0.9397	-	
		Ctrl 24 x AD 24	-	-	0.8150	-	
		Age	7.1389	2,29	0.0037	**	

	L-Glutamic Acid	ANOVA	Genotype	6.5564	1,29	0.0172	*	6i
			Age x Genotype	3.9331	2,29	0.0333	*	
		Tukey HSD	Ctrl 6 x Ctrl 12	-	-	0.0028	**	
			Ctrl 6 x Ctrl 24	-	-	0.5212	-	
			Ctrl 12 x Ctrl 24	-	-	0.3769	-	
			AD 6 x AD 12	-	-	0.9407	-	
			AD 6 x AD 24	-	-	0.3796	-	
			AD 12 x AD 24	-	-	0.1623	-	
			Ctrl 6 x AD 6	-	-	0.0041	**	
			Ctrl 12 x AD 12	-	-	0.8962	-	
			Ctrl 24 x AD 24	-	-	1.0000	-	
	Pyruvate	ANOVA	Age	0.3854	2,29	0.6843	-	6j
			Genotype	5.7967	1,29	0.0241	*	
			Age x Genotype	3.3749	2,29	0.0511	-	
		Tukey HSD	Ctrl 6 x AD 6	-	-	0.0105	*	
			Ctrl 12 x AD 12	-	-	0.1940		
			Ctrl 24 x AD 24	-	-	0.7330		
	Uracil	ANOVA	Age	2.5451	2,29	0.0994	-	6k
			Genotype	10.2997	1,29	0.0038	**	
			Age x Genotype	1.8240	2,29	0.1830	-	
		Tukey HSD	Ctrl 6 x AD 6	-	-	0.0110	*	
			Ctrl 12 x AD 12	-	-	0.4853	-	
			Ctrl 24 x AD 24	-	-	0.9960	-	
	7-Methylguanine	ANOVA	Age	5.5281	2,29	0.0106	*	6l
			Genotype	6.1897	1,29	0.0202	*	
			Age x Genotype	1.4904	2,29	0.2454	-	
		Tukey HSD	Ctrl 6 x Ctrl 12	-	-	0.9989	-	
			Ctrl 6 x Ctrl 24	-	-	0.0454	*	
			Ctrl 12 x Ctrl 24	-	-	0.0642	-	
			AD 6 x AD 12	-	-	0.7806	-	
			AD 6 x AD 24	-	-	0.9764	-	
			AD 12 x AD 24	-	-	0.5089	-	
			Ctrl 6 x AD 6	-	-	0.0391	*	
			Ctrl 12 x AD 12	-	-	0.8874	-	
Spleen			Ctrl 24 x AD 24	-	-	0.9975	-	
		ANOVA	Age	0.9135	2,29	0.4146	-	
			Genotype	0.0627	1,29	0.8044	-	
			Age x Genotype	11.2878	2,29	0.0004	**	

	Isovalerylcarnitine	Tukey HSD	Ctrl 6 x Ctrl 12	-	-	0.0006	**	6m
			Ctrl 6 x Ctrl 24	-	-	0.3175	-	
			Ctrl 12 x Ctrl 24	-	-	0.2921	-	
			AD 6 x AD 12	-	-	0.3139	-	
			AD 6 x AD 24	-	-	0.6803	-	
			AD 12 x AD 24	-	-	0.9933	-	
			Ctrl 6 x AD 6	-	-	0.0067	**	
			Ctrl 12 x AD 12	-	-	0.0949	-	
			Ctrl 24 x AD 24	-	-	1.0000	-	
	2-Methylbutyroylcarnitine	ANOVA	Age	1.3931	2,29	0.2677	-	6n
			Genotype	0.0591	1,29	0.8101	-	
			Age x Genotype	8.2213	2,29	0.0019	**	
		Tukey HSD	Ctrl 6 x Ctrl 12	-	-	0.0045	**	
			Ctrl 6 x Ctrl 24	-	-	0.9713	-	
			Ctrl 12 x Ctrl 24	-	-	0.0847	-	
			AD 6 x AD 12	-	-	0.4097	-	
			AD 6 x AD 24	-	-	0.4443	-	
			AD 12 x AD 24	-	-	1.0000	-	
			Ctrl 6 x AD 6	-	-	0.0326	*	
			Ctrl 12 x AD 12	-	-	0.1615	-	
			Ctrl 24 x AD 24	-	-	1.0000	-	
	n6-Acetyl L-Lysine	ANOVA	Age	6.5032	2,29	0.0055	**	6o
			Genotype	4.5612	1,29	0.0431	*	
			Age x Genotype	3.7111	2,29	0.0394	*	
		Tukey HSD	Ctrl 6 x Ctrl 12	-	-	0.9923	-	
			Ctrl 6 x Ctrl 24	-	-	0.9766	-	
			Ctrl 12 x Ctrl 24	-	-	0.9998	-	
			AD 6 x AD 12	-	-	0.9773	-	
			AD 6 x AD 24	-	-	0.0041	**	
			AD 12 x AD 24	-	-	0.0628	-	
			Ctrl 6 x AD 6	-	-	0.0615	-	
			Ctrl 12 x AD 12	-	-	0.2521	-	
			Ctrl 24 x AD 24	-	-	0.9196	-	
		ANOVA	Age	1.8797	2,29	0.1744	-	
			Genotype	0.2122	1,29	0.6492	-	
			Age x Genotype	4.4834	2,29	0.0222	-	
			Ctrl 6 x Ctrl 12	-	-	0.2615	-	
			Ctrl 6 x Ctrl 24	-	-	0.4271	-	

Dimethylglycine	*Tukey HSD*	Ctrl 12 x Ctrl 24	-	-	1.0000	-	6p
		AD 6 x AD 12	-	-	0.9998	-	
		AD 6 x AD 24	-	-	0.2244	-	
		AD 12 x AD 24	-	-	0.2547	-	
		Ctrl 6 x AD 6	-	-	0.1489	-	
		Ctrl 12 x AD 12	-	-	0.9824	-	
		Ctrl 24 x AD 24	-	-	0.4695	-	
Hydroxyproline	ANOVA	Age	0.4343	2,29	0.6527	-	6q
		Genotype	0.1619	1,29	0.6910	-	
		Age x Genotype	4.5728	2,29	0.0208	*	
	Tukey HSD	Ctrl 6 x Ctrl 12	-	-	0.2787	-	
		Ctrl 6 x Ctrl 24	-	-	0.3634	-	
		Ctrl 12 x Ctrl 24	-	-	1.0000	-	
		AD 6 x AD 12	-	-	0.9786	-	
		AD 6 x AD 24	-	-	0.3198	-	
		AD 12 x AD 24	-	-	0.8311	-	
		Ctrl 6 x AD 6	-	-	0.0823	-	
		Ctrl 12 x AD 12	-	-	1.0000	-	
		Ctrl 24 x AD 24	-	-	0.6733	-	
Azelaic Acid	ANOVA	Age	0.3265	2,29	0.7246	-	
		Genotype	12.6179	1,29	0.0016	**	6r
		Age x Genotype	14.1810	2,29	0.0001	***	
	Tukey HSD	Ctrl 6 x Ctrl 12	-	-	0.0010	**	
		Ctrl 6 x Ctrl 24	-	-	0.0425	*	
		Ctrl 12 x Ctrl 24	-	-	0.9637	-	
		AD 6 x AD 12	-	-	0.1384	-	
		AD 6 x AD 24	-	-	0.2409	-	
		AD 12 x AD 24	-	-	0.9998	-	
		Ctrl 6 x AD 6	-	-	0.0001	***	
		Ctrl 12 x AD 12	-	-	0.9973	-	
		Ctrl 24 x AD 24	-	-	0.9942	-	
Glycerate	ANOVA	Age	0.4616	2,29	0.6358	-	6s
		Genotype	15.7548	1,29	0.0006	**	
		Age x Genotype	4.0682	2,29	0.0301	*	
		Ctrl 6 x Ctrl 12	-	-	0.9924	-	
		Ctrl 6 x Ctrl 24	-	-	0.6626	-	
		Ctrl 12 x Ctrl 24	-	-	0.8799	-	
		AD 6 x AD 12	-	-	0.6924	-	

		Tukey HSD	AD 6 x AD 24	-	-	0.1822	-	
			AD 12 x AD 24	-	-	0.9545	-	
			Ctrl 6 x AD 6	-	-	0.0005	**	
			Ctrl 12 x AD 12	-	-	0.2308	-	
			Ctrl 24 x AD 24	-	-	0.9998	-	
	1-Methylnicotinamide	ANOVA	Age	1.2360	2,29	0.3084	-	6t
			Genotype	4.7186	1,29	0.0399	*	
			Age x Genotype	6.4571	2,29	0.0057	**	
		Tukey HSD	Ctrl 6 x Ctrl 12	-	-	0.3677	-	
			Ctrl 6 x Ctrl 24	-	-	0.9554	-	
			Ctrl 12 x Ctrl 24	-	-	0.9483	-	
			AD 6 x AD 12	-	-	0.1511	-	
			AD 6 x AD 24	-	-	0.0778	-	
			AD 12 x AD 24	-	-	0.9997	-	
			Ctrl 6 x AD 6	-	-	0.0013	**	
			Ctrl 12 x AD 12	-	-	0.9996	-	
			Ctrl 24 x AD 24	-	-	1.0000	-	
	Sphingosine-1-phospate	ANOVA	Age	2.0079	2,29	0.1562	-	6u
			Genotype	1.4813	1,29	0.2354	-	
			Age x Genotype	6.2495	2,29	0.0065	**	
		Tukey HSD	Ctrl 6 x Ctrl 12	-	-	1.0000	-	
			Ctrl 6 x Ctrl 24	-	-	0.7866	-	
			Ctrl 12 x Ctrl 24	-	-	0.7735	-	
			AD 6 x AD 12	-	-	0.3867	-	
			AD 6 x AD 24	-	-	0.0161	*	
			AD 12 x AD 24	-	-	0.7219	-	
			Ctrl 6 x AD 6	-	-	0.0162	*	
			Ctrl 12 x AD 12	-	-	0.9243	-	
			Ctrl 24 x AD 24	-	-	0.5419	-	
